# Unveiling the third dimension in morphometry with automated quantitative volumetric computations

**DOI:** 10.1038/s41598-021-93490-4

**Published:** 2021-07-14

**Authors:** Lawrence R. Frank, Timothy B. Rowe, Doug M. Boyer, Lawrence M. Witmer, Vitaly L. Galinsky

**Affiliations:** 1 Institute for Engineering in Medicine, Center for Scientific Computation in Imaging, University of California San Diego, 8950 Villa La Jolla Dr., Suite B227, La Jolla, CA 92037 USA; 2 Department of Radiology, Center for Functional MRI, University of California San Diego, 9500 Gilman Dr., #0677, La Jolla, CA 92093-0677 USA; 3 Department of Geological Sciences, Jackson School of Geosciences, University of Texas, Austin, TX 78712 USA; 4 Department of Evolutionary Anthropology, Duke University, Chapel Hill, NC USA; 5 Department of Biomedical Sciences, Heritage College of Osteopathic Medicine, Ohio University, Athens, OH USA

**Keywords:** Software, Applied mathematics, Computational science, Palaeontology, Evolutionary theory, Software, Computational platforms and environments, Image processing, Data processing

## Abstract

As computed tomography and related technologies have become mainstream tools across a broad range of scientific applications, each new generation of instrumentation produces larger volumes of more-complex 3D data. Lagging behind are step-wise improvements in computational methods to rapidly analyze these new large, complex datasets. Here we describe novel computational methods to capture and quantify volumetric information, and to efficiently characterize and compare shape volumes. It is based on innovative theoretical and computational reformulation of volumetric computing. It consists of two theoretical constructs and their numerical implementation: the spherical wave decomposition (**SWD**), that provides fast, accurate automated characterization of shapes embedded within complex 3D datasets; and symplectomorphic registration with phase space regularization by entropy spectrum pathways (**SYMREG**), that is a non-linear volumetric registration method that allows homologous structures to be correctly warped to each other or a common template for comparison. Together, these constitute the Shape Analysis for Phenomics from Imaging Data (**SAPID**) method. We demonstrate its ability to automatically provide rapid quantitative segmentation and characterization of single unique datasets, and both inter-and intra-specific comparative analyses. We go beyond pairwise comparisons and analyze collections of samples from 3D data repositories, highlighting the magnified potential our method has when applied to data collections. We discuss the potential of ***SAPID*** in the broader context of generating normative morphologies required for meaningfully quantifying and comparing variations in complex 3D anatomical structures and systems.

## Introduction

### Imaging and data complexity

Advances in modern digital imaging methods are revolutionizing a wide range of scientific disciplines by facilitating the acquisition of huge amounts of 3D volumetric data that represent objects of scientific interest with unprecedented fidelity. Such data can often replace examination of physical objects and thereby increase the quantitative rigor and computational power that can be aimed at the analysis of those objects^1^. This is particularly true in the fields of evolutionary biology and paleontology where 3D volume data avail both the external and internal complex geometry of museum specimens to detailed visualization and quantitative study. In addition, digitizing and virtualizing research objects such as those in museum collections allows them to be deposited in web repositories or broadly shared, thereby increasing accessibility and value of these collections for research and public benefit, while simultaneously reducing the risk of damage to fragile specimens due to handling. Methods such as MRI and CT are rapidly changing the world of evolutionary biology research and have had a profound influence on scientific discovery. These data are just the starting point for the scientific exploration that modern computational and visualization methods enable. In particular, our ability to understand phenomic effects of genetic variation is limited by availability of quantitative representations of the morphological/anatomical phenomes in this case). Therefore, the more advanced our ability to characterize morphological variation, the more we can eventually understand about genetic variation.

These advances come at a cost. While the reservoir of digitized specimen data is growing rapidly, as evidenced from the frequency of published papers using digital volume data^2–14^, the cumulative impact and power of these data remains unrealized because of the labor intensive data analysis. As dataset size, dimensionality and complexity grow along with sample sizes (number of datasets available), so does the need for increasingly efficient and accurate algorithms capable of robustly extracting quantitative parameters relevant to research questions. The difficulties are not only computational, as data sizes and dimensionalities continue to increase, but also, perhaps surprisingly, theoretical as well: The vast majority of studies still rely on surfaced based analysis methods, and thus do not fully exploit the volumetric nature of the data and the vast amount of information it contains.

In short, the great potential that these advanced digital imaging systems have for making sense of biological diversity remains untested in many ways because of the significant difficulties in analyzing the increasingly complex volumes of data.

### Phenomics and computational morphology

During the past decade, biology has been revolutionized by enormous advances in molecular and cellular biology. Conspicuously, comparative morphology has been slower to evolve during this same period^15^, even though great technological advances in imaging can provide volumetric digital data of exquisite resolution. One reason why morphometrics has lagged behind genomics is the difficulty in characterizing and comparing complex morphological variations in 3D volumetric data^16^. Moreover, volumetric morphological datasets are also orders of magnitude larger than sequence data.

Broadly speaking, revitalizing the study of anatomical aspects of organismal phenome (i.e., morphology) is critical, as patterns of shape variation in biological structures form the basis for understanding important aspects of gene function^17,18^, developmental mechanisms^19^, ecological adaptation^20,21^, and evolutionary history^22^. This is also an important short-fall because as research designs in phylogenetics become increasingly multi-factorial and complex, there is an increased need for more accurate and sophisticated techniques to measure morphological variation, particularly as modern scanners provide increasing resolving power. Further—though amazing insight continues to flow from rapid and profound advances in genomics—there is growing recognition that the utility and information content of genetic data will only reach its fullest extent once data on associated phenotypes can be analyzed at equivalent rates and scales^23^. This is a central issue for the emerging field of phenomics^24^.

Geometric morphometrics has emerged as an important tool for phenomics, becoming commonly used to quantify morphology, wherein landmark points are identified in images and then are fit to a warped mesh that provides a common coordinate system in which different specimens can be compare^25–32^. Landmark coordinates are the basis of statistical comparisons of geometric morphometrics. These coordinates are intended to be biologically equivalent on all specimens analyzed and they are usually defined manually, though more recently new algorithmic approaches for automatically placing landmarks have begun to arise^16,33–36^ and statistically compare morphologies based on these points. However, current methods remain primarily surface-based^37–41^. In fact, true volumetric analysis should be much better suited to detecting and comparing subtle morphological variations and is a more efficient use of volumetric data. But the extension to volumetric analysis comes at the cost of an increased complexity of the analysis.

### Shape analysis for phenomics from imaging data (SAPID)

Over the last several years, our (Dr Frank and Dr Galinsky) *Center for Scientific Computation in Imaging* (CSCI, http://csci.ucsd.edu) has developed a theoretical and computational methodology to address the two major technological issues involved in the application of shape analysis to 3D volumetric data to phenomics: (1) *characterizing* and (2) *comparing* shapes. Characterizing volumetric geometric properties of different tissue types within a sample involves **shape analysis** and **image segmentation** and is done on single specimens, while comparing geometric characteristics involves **image registration** and is done between specimens. The two algorithms we have developed for addressing these issues are the Spherical Wave Decomposition (**SWD**)^42^ for shape analysis and image segmentation, and *symplectomorphic registration with phase space regularization by ESP* (**SYMREG**)^43^ for registration. Together, we collectively call these the *Shape Analysis for Phenomics from Imaging Data* (SAPID) toolkit.

The technical details of these methods have been discussed elsewhere^42,43^, so here we will first present the methodology in more descriptive and intuitive form. Our main objective in this paper though is to describe the application of this methodology to different volumetric datasets from three collaborators (Drs Rowe, Boyer, and Witmer) who make heavy use of imaging in their ongoing research in evolutionary biology and paleontology. The intent is to show that this small sample of data from a broad spectrum of applications will serve as sufficient demonstration of the practical utility of our approach in solving computational morphology problems of widespread applicability. Data used in this study are shown in Table 1.

The theoretical foundations and computational implementations of the SAPID methods SWD and SYMREG have been described in detail in previous publications^42,43^. These papers are necessarily quite technical in order to describe the details of the algorithm implementations. Here we will focus on a more intuitive description of the methods with some illustrative examples aimed at elucidating the important features of the methods.

## Results

### Example 1: analysis of CT growth series data of stained *Monodelphis* specimens (Rowe Lab)

In the late 1980s and early 90s Dr Rowe’s team assembled a calibrated growth series of the opossum *Monodelphis domestica*, a popular experimental model species for early-diverging mammals. It was provided by the Southwestern Foundation for Biomedical Research, an NIH-sponsored facility that supports research on model organisms. The collection consists of $\approx 200$ specimens some of which were cleared and double-stained, and others skeletonized. Six of the small skulls representing different levels of maturity (post-natal days 27, 48, 57, 75, 90, and a retired breeder) were the first to be CT scanned using an industrial high-resolution scanner at the University of Texas High-Resolution X-ray Computed Tomography Facility (UTCT). At that time it was a state-of-the-art instrument, producing $512 \times 512$ pixel images representing $200\upmu$ thick slices, and 300 Mb datasets (see DigiMorph.org). Early research on these data provided novel insights into the effects of relative growth of the brain and auditory ossicles including a novel solution to the problem of evolution of the mammalian middle ear. It took weeks to manually register images from the different datasets, to plot growth vectors for different elements of the braincase and middle ear, and to illustrate the results which were sufficiently important for publication in the journal Science^44^.

**Figure 1:**
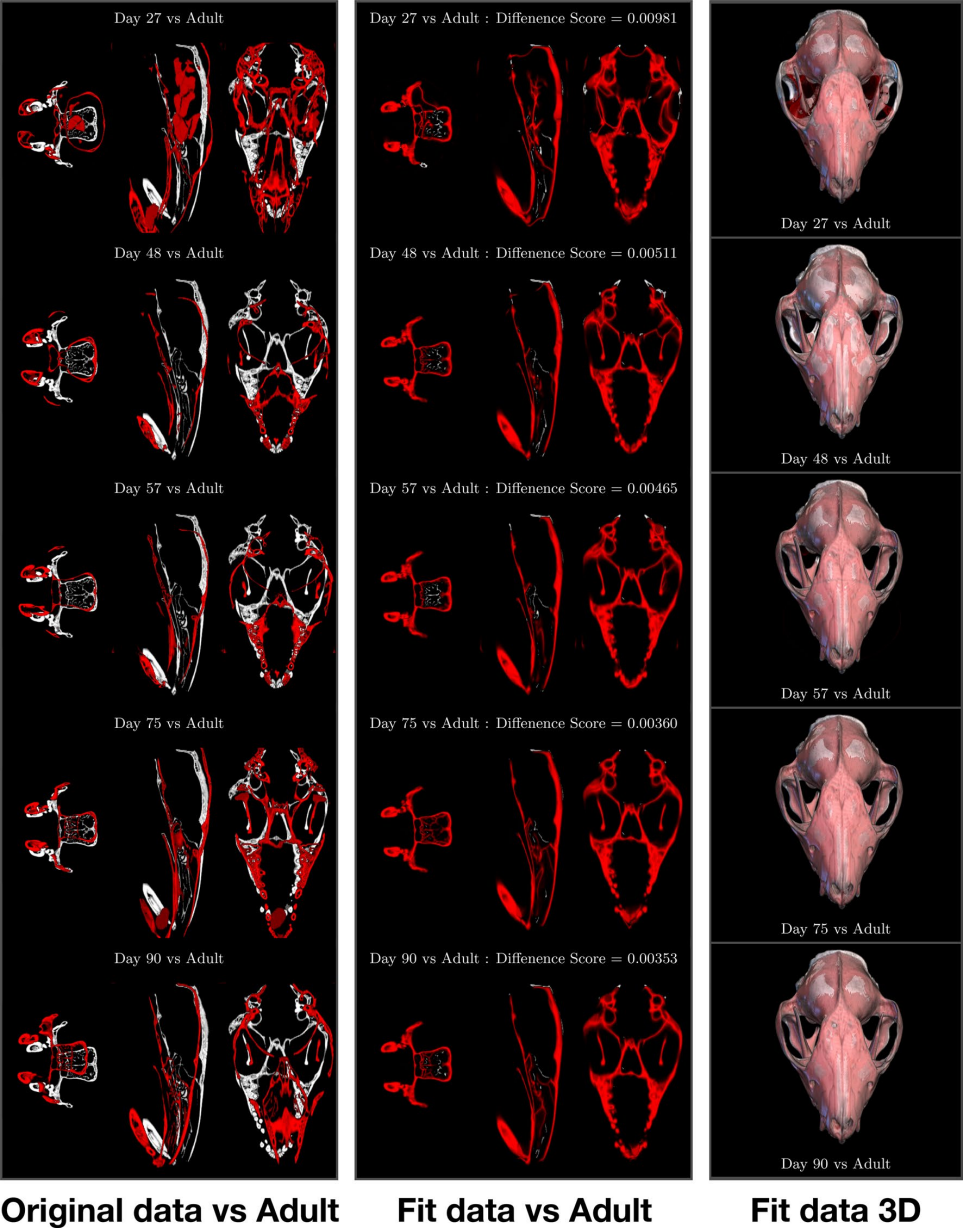
Volumetric SAPID analysis of high resolution X-ray CT images of Monodelphis. (Left) Slice through original volumetric CT data (red) overlayed with adult CT (grayscale), (Middle) Slice through volumetric non-linear registration of original CT data (red) overlayed with adult CT (grayscale), (Right) 3D visualization of volumetric non-linear registration of original CT data (red) overlayed with adult CT (grayscale).

Applying **SAPID** to register pairs of these same datasets took only a few hours and required no user manual input (Fig. 1). The results not only corroborated the earlier findings of differential growth trajectories for specific points on the skull, but also provided comparisons of the entire skulls in different slice planes that reveal differential growth in the face and dentition that have not previously been studied quantitatively in 3D. It also enabled identification of artifacts resulting from drying the skulls before scanning, especially in the less mature specimens. Preparation artifacts are rarely identified as sources of uncertainty in morphometric comparisons between recent specimens. With our latest Northstar ultra high-resolution scanner, we can generate imagery up to $4098 \times 4098$ pixels, at micron-scale resolutions, and data volumes more than an order of magnitude larger than those produced by the previous generations of scanners. The advent of iodine-staining and phase contrast scanning^45^ has enabled CT to image soft tissues as well as the skeleton. CT is now poised to facilitate a much fuller understanding of the complex dynamic between the developing skull, its neurosensory structures, and its musculature. Moreover, we can now successfully image 1-day-old specimens in which nearly all of the skeleton is still cartilaginous, and capture all of postnatal ontogeny using a much denser sample of specimens. The data volumes will quickly grow into hundreds of gigabytes. Quantitative analysis and visualization of such large and rich volumes of data can for the first time be accomplished using the improved, automated algorithms integrated in *SAPID*.

Additionally, *SAPID* provided the SWD quantitative geometric measures that compares skull shape between specimens. The original data is shown in Fig. 1(left) and the registered data is shown in Fig. 1(middle). We emphasize that these results are fully volumetric, as shown in Fig. 1(right). The measurements indicate rapid differentiation of shape in early ontogeny followed by slowing and then a plateau approaching maturity. These results are not surprising, but quantifying rates of morphogenesis over the course of ontogeny has been limited to surface based methods until now, which limits the ability to detect more subtle morphological changes that occur in 3D. Using fossils in a phylogenetic context, we will be able to quantify rates of evolutionary morphogenesis througout the entire image volume as well. Whole head analyses, quantitatively in 3D, will enable testing of recent hypotheses that olfactory gene duplications were major drivers in the evolution of skull and cortical organization along the mammalian stem and in many of its living clades^46,47^.

### Example 2: posture correction and interspecies skull comparison (Boyer Lab)

It has long been recognized size variation among individuals within a species scales proportionally and is generally less than size variation between species^48^. Understanding of the intraspecific limits on size variation has been critical for utilizing data on size variation to address questions about evolution. A complete geometric characterization not just of size but shape necessarily provides greater quantitative sensitivity to evolutionary changes. Classic morphometric methods have proven useful in this problem but are limited by their restriction to surfaces, and not easy to generalize to full volumetric data. They are also somewhat limited in the application to specimens that have well defined similar landmarks, though even in this case they artificially assume the importance of certain landmarks chosen a priori, as discussed below in “Hypothesis testing” section.

In the case where similar landmarks do not exist, current analysis methods become even more problematic. However, the volumetric registration of SAPID is unaffected by this problem, as no landmark identification is required a priori. Even in the absence of similar landmarks, SAPID provides a general method for describing intraspecific variation in different species by iteratively permuting monospecific sample compositions to generate a distribution of best-parameters for each sample. The distribution of SWD parameters then becomes the measure of shape variation that can be compared between species. When pairs of groups have non-overlapping distributions of parameters, one could define these groups as having heterogeneous levels of variation. The same parameters that we use to demonstrate differences in intraspecific variation can also be used to assess group membership of unknown individuals or to assess shape overlap between populations.

**Figure 2:**
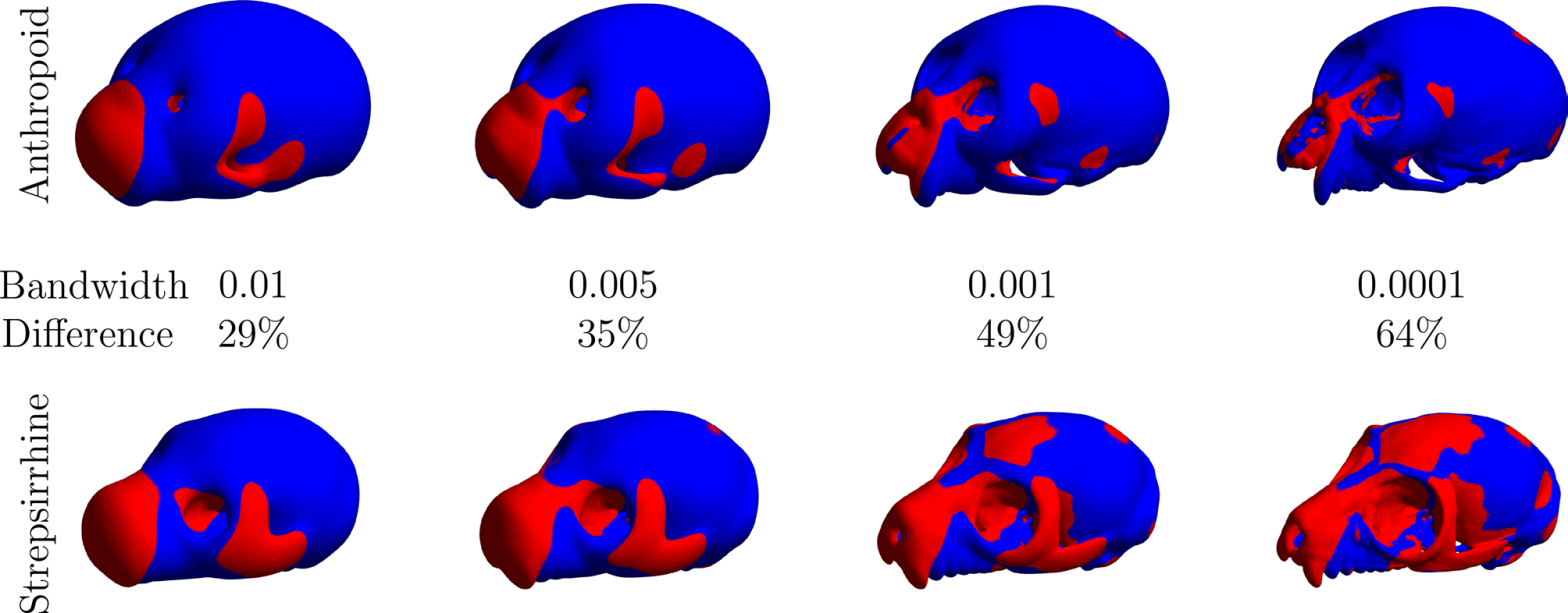
Skull shape characterization by Spherical Wave Decomposition (SWD) on skulls of an Anthropoid and a Strepsirrhine from MorphoSource. Bandwidth (i.e. more accurate shape description) increases from left to right. Blue voxels are similar between skulls, red are dissimilar. As fit increases, the percent of voxels in the volume that are different between the species increases.

**Figure 3:**
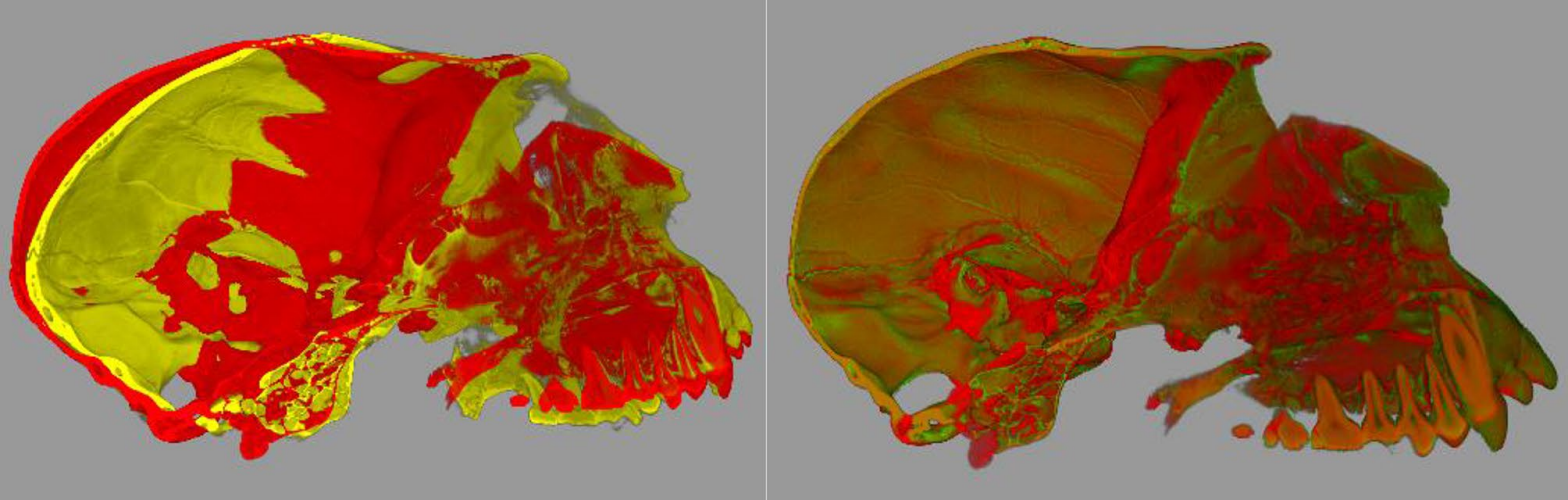
CT scans of two *Callicebus cupreus* skulls archived in MorphoSource. (Left) original and (right) registered using symplectomorphic non-linear registration. The yellow is registered to the red. Red is the “template”, yellow is warped to the “template” space.

**Figure 4:**
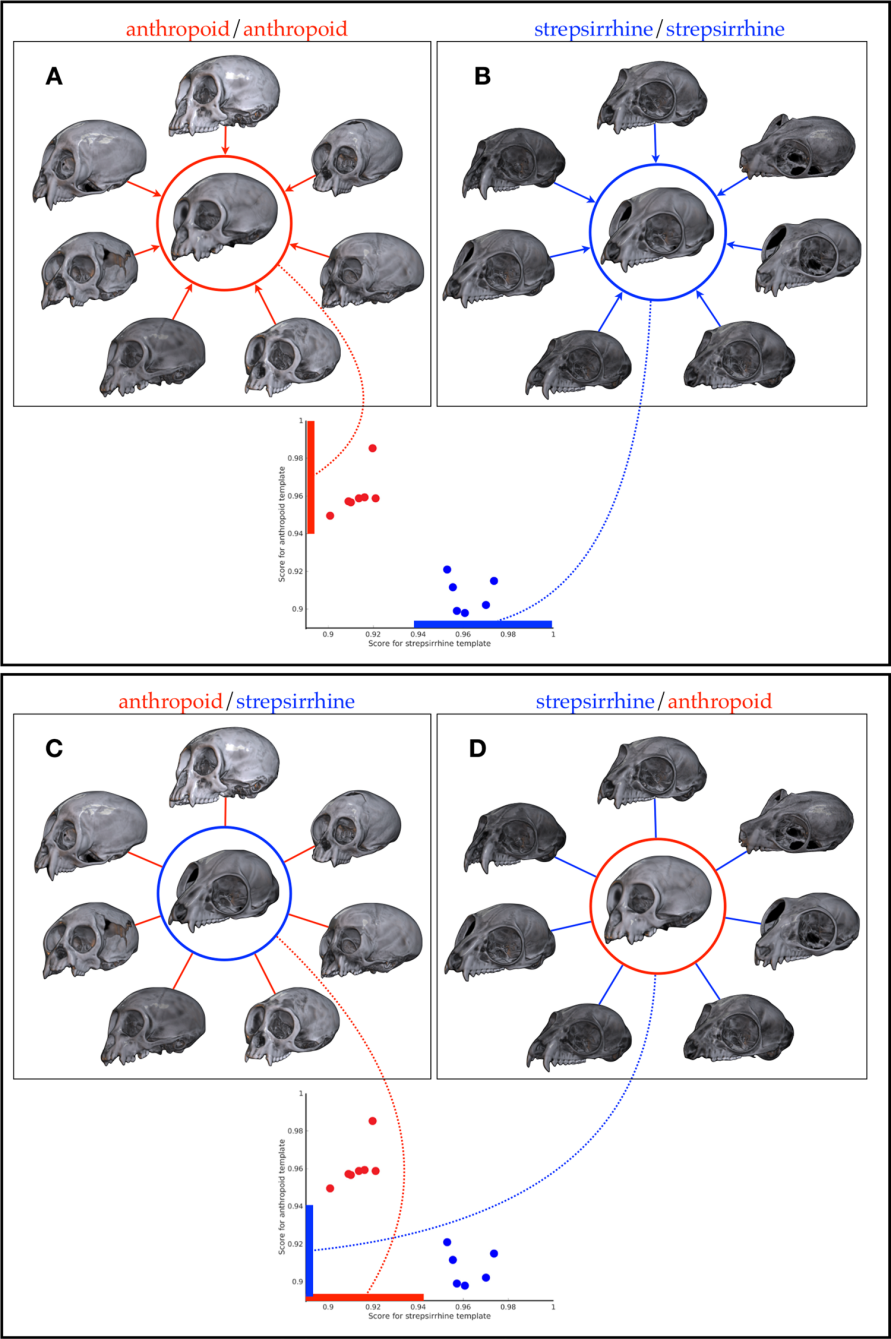
An example of intra-species (**A**, **B**) and inter-species (**C**, **D**) quantitative morphological comparison using CT scans of seven anthropoid and seven strepsirrhine skulls. **Intra-species comparison.** (**A**) Red circle encloses average anthropoid skull computed from the surrounding 7 individual skulls using SYMREG. Similarity values for individual skulls compared with the template is show as the red dot on the plot with scores on the vertical axis. (**B**) Blue circle encloses average strepsirrhine skull computed from the surrounding 7 individual skulls using SYMREG. Similarity values for individual skulls compared with the template is show as the blue dot on the plot with scores on the horizontal axis. Scores are high in all cases, as would be expected, and suggest each skull is from the same species. Variations provided by variations from the mean form a basis for quantitative comparision. **Inter-species comparison.** In (**C**) the average strepsirrhine from (**B**) is used for the comparison template for the seven anthropoid skulls in (**A**). The similarity values (pointed to by red dotted line) are all low, suggesting each is a poor match in species. In (**D**) the average anthropoid from (**A**) is used for the comparison template for the seven strepsirrhine skulls in (**B**). Again, the poor similarity scores (pointed to be blue dotted line) suggest that the species are different. Note that while the similarity measure here (mean squared difference) is a simple scalar values, it is derived from the *volumetric* differences between individual skulls and templates which can be used to identify specific locations of geometric variations.

A demonstration of the capabilities of shape characterization with SWD is shown in Fig. 2 where skulls from an anthropoid and a strepsirrhine (from MorphoSource.org) are compared by characterizing each in terms of the SWD at different accuracies which allows the quantitative characterization of similar and dissimilar local and global shape changes. A demonstration of volumetric non-linear registration using SYM-REG on volumetric CT scans of two *Callicebus cupreus* skulls is shown in Fig. 3. An example of intra-species and inter-species quantitative morphological comparison facilitated by SAPID is shown in Fig. 4 using volumetric CT scans of quantitative morphological comparison using CT scans of seven anthropoid skull and seven strepsirrhine skulls.

**Figure 5:**
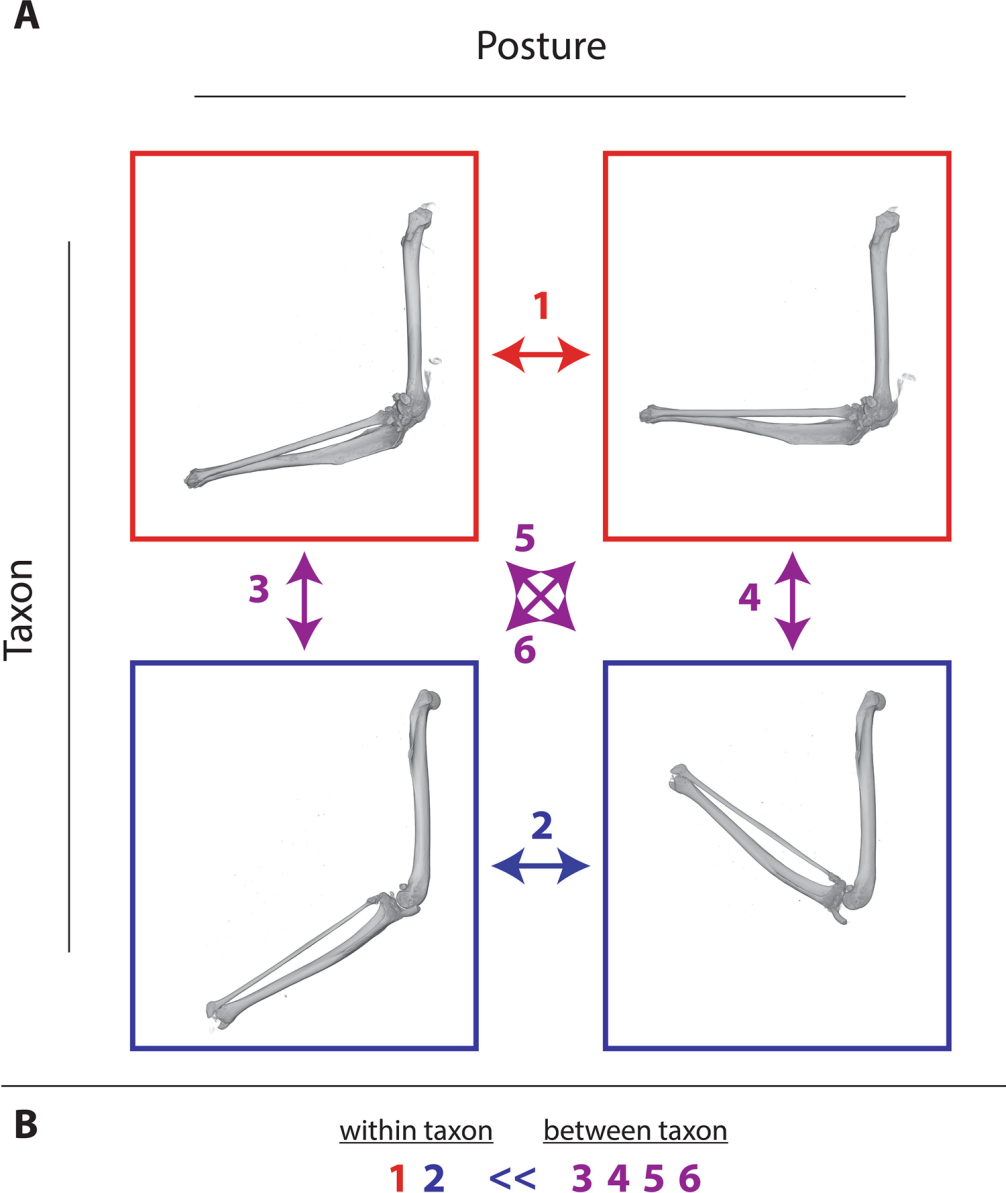
SAPID allows postural differences to be excluded from shape comparisons. (**A**) The same structure (a mammal hindlimb—femur and tibia/fibula) was scanned twice (boxes of the same color) in two different postures (a flexed and extended posture). Then scans of the same structure in different postures were compared to each other using SAPID (#1–2). As well, all combinations of different specimens in similar and different postures were compared (#3–6). (**B**) Despite different postures, SAPID successfully identified instances of the same structure in different postures as morphologically more similar (having smaller computed morphological distances) than comparisons of different structures. This suggests that whole body scans can be compared to quantify interspecific differences in body plan even though each specimen that is scanned has a unique posture. Red box = primate limb, Blue box = treeshrew limb.

These whole body datasets present a more difficult but very critical practical problem for characterizing phenotypic differences. Variations of posture of different specimens introduce large differences in volumetric geometry unrelated to differences due to shape of individual bones. We found that SAPID is able to distinguish these, as demonstrated by the example where the same specimen scanned twice in two completely different postures can still be recognized as uniquely similar even when compared to different specimens in similar postures (Fig. 5). In addition, the SAPID derived parameters have been able to demonstrate the relative similarity of different scans. This is completely outside the realm of capability of any other shape comparison method based on either volumes or surfaces.

### Example 3: shape analysis and segmentation of *Tyrannosaurus rex* (Witmer Lab)

Paleobiology is another field where the rapid advancement of imaging and computational capabilities is completely altering our understanding of form and function. The SAPID algorithms have the capabilities of addressing common problems in vertebrate paleontology, such as deformation, disarticulation, and missing bony elements, within a framework of rigor and repeatability, and help address key questions in dinosaur paleobiology.

Imaging in paleobiology research shares common goals with evolutionary biology of extant species, such as automatic segmentation and quantitative shape characterization of internal structures and statistical assessment of structure variations between specimens. But extending these analytical advances into deep time, however, presents challenges for paleobiological interpretation due to the added complication of diagenetic and taphonomic factors.

**Figure 6:**
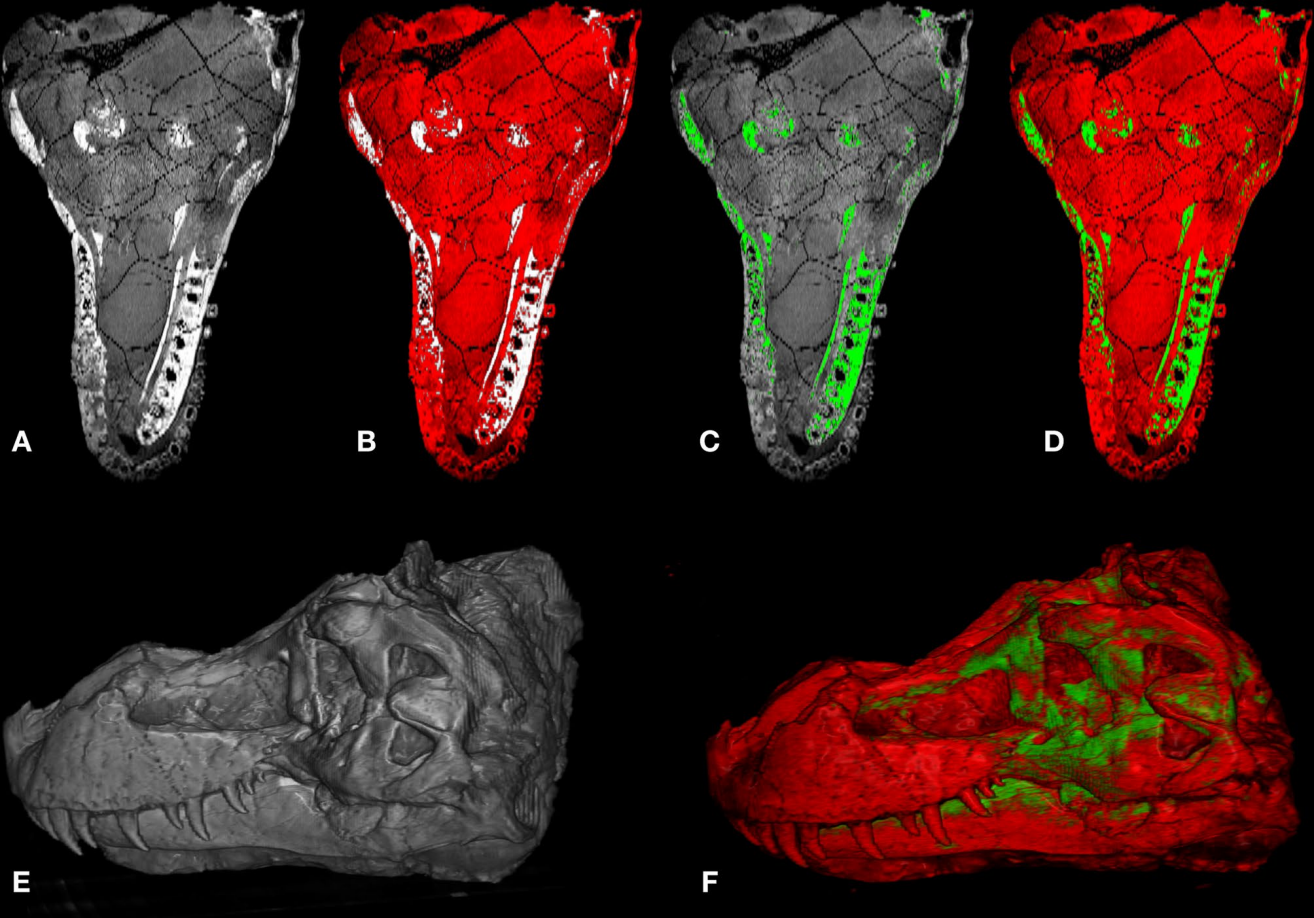
Automated shape analysis and segmentation of Tyrannosaurus Sue skull. (**A**) Slice through original CT volumetric data; (**B**) SWD of low intensity values, (**C**) SWD of high intensity values; (**D**) combined display of (**B**, **C**); (**E**) unsegmented SWD reconstruction of entire volume, (**F**) combined segmented SWD volumes.

Ultimately, optimization of the SAPID methods for paleobiological data will require addressing the unique characteristics of CT in inhomogeneous samples (e.g., fossils in rock matrix, often with additional materials introduced by museum staff such as plaster, adhesives, and metal mounting armature) and the optimal fitting parameters for data that contain a large number of sharp edges (e.g., from fractures). We performed an initial SWD analysis on the skull of the well-known specimen of *Tyrannosaurus rex* known as “Sue” (FMNH PR 2081). This specimen, on display at the Field Museum of Natural History in Chicago, is among the most complete (approximately 85%) and largest specimens of *T. rex* collected. The results, shown in Fig. 6, demonstrate the ability to fit and segment a specimen that has these issues.

Future work will require addressing deformation and other taphonomic artifacts, including development of standardized analysis and reproducibility pathways for retrodeformation of fossils, and optimizing the tools to take into account diagenetic and/or taphonomic deformation, which hampers analytical comparison of specimens.

Just as it is able to factor out differences due to posture, the SAPID tools also have the potential to meaningfully quantify and compare specimens even when bones are out of position or missing, as is often the case in fossils. This will provide a level of objectivity and repeatability previously lacking in dinosaur studies.

The ultimate goal of SAPID in this application is the elucidation of specific paleobiological issues such as dinosaur skull function, such as sensory organization and behavior of tryannosaurs^49^. Analytical advances in bioengineering—such as finite element analysis (FEA) of feeding mechanics or computational fluid dynamics (CFD) of nasal airflow—are highly sensitive to structural conformation. Having powerful analytical software connected to high-fidelity 3D data for multiple specimens within different clades will allow unprecedented computational accuracy and reproducibility of functional studies. In conjunction with soft-tissue reconstructions (e.g., jaw muscles, brain), this will allow hypothesis-testing of critical functional systems (e.g., feeding, respiration, sound production, sensory and cognitive ability).

### Example 4: the fossil problem: *Archaeoptery × lithographica* (Rowe Lab)

**Figure 7:**
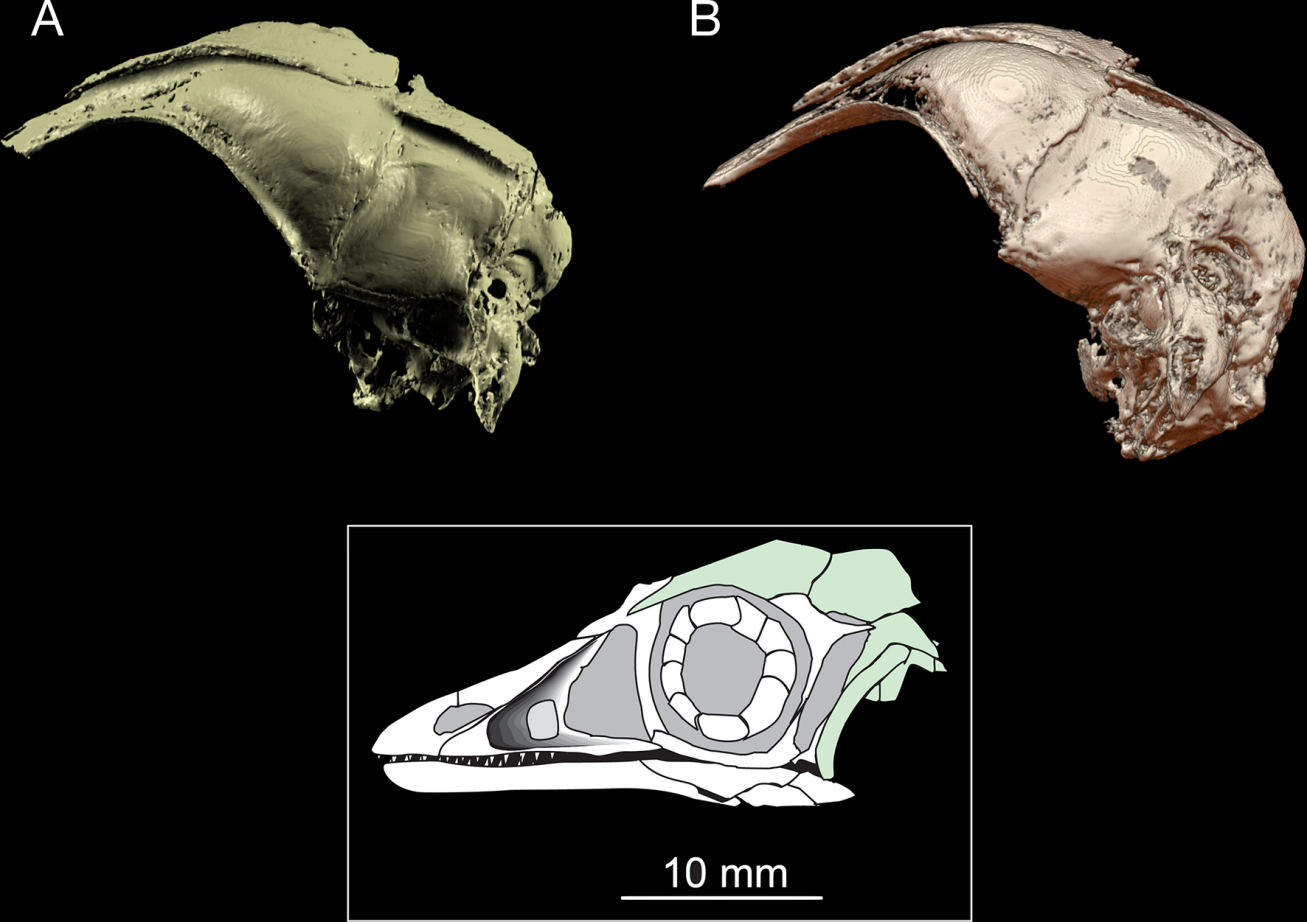
Shape analysis and segmentation of a CT scan of the skull of the London specimen (BMNH 37001) of *Archaeopteryx lithographica* for a study of the brain and inner ear and its adaptation to flight^50^. Specimen was scanned at UT Austin’s High-Resolution X-ray CT Facility at low and high X-ray energies (120 kV and 180 kV). The resulting matrix data size was $1024 \times 1024 \times 650$ voxels and the voxel size was $20 \times 20 \times 46\, \upmu {\mathrm{m}}$. (**A**) The original segmentation (Fig. 1b in^50^) which took ${\sim }\,100$ h of manual segmentation. (Visualization using Mimics v7.3, https://www.materialise.com) (**B**) Automated SWD analysis. The SWD detects continuous distributions of similar intensity and quantitatively characterizes their shape. Subsequent to the SWD analysis, a segmentation algorithm extracts the skull. The total processing time to produce figure (**B**) was 7 min and required no user input.

A good example of the challenges facing paleobiological imaging data, and the potential utility of SAPID in addressing them, is illustrated with high resolution CT data of the skull of the important London specimen of *Archaeopteryx lithographica* (BMNH 37001) shown in Fig. 7. This was the subject of a study that elucidated the avian nature of the brain and inner ear and its adaptation to flight^50^.

The braincase of BMNH 37001 was scanned twice, at low and high X-ray energies (120 kV and 180 kV, respectively) The resulting matrix data size was $1024 \times 1024 \times 650$ voxels and the voxel size was $20 \times 20 \times 46 \upmu {\mathrm{m}}$. (For full scanning details, see^50^, and additioanal imagery on http://digimorph.org/specimens/Archaeopteryx_lithographica/).

In Fig. 7A is shown the results of the original manual processing (Fig. 1b in^50^) using Mimics v7.3 software (www.materialise.com). This required a significant amount of hand editing and took approximate 100 h to complete. In Fig. 7B is shown the SWD analysis, which used on the high power (180 kV) data. The computation of the SWD took $\approx 2$ min and the segmentation took $\approx 5$ mins.

It is important to recognize that there is a fundamental difference between the traditional histogram method and the SWD method for segmentation. The most principled approach to histogram based segmentation is to estimate different statistical distributions of intensities. However, this will still include *any* voxels with similar intensities, even if they are spatially isolated from the large scale structures, such as the ubiquitous sand grains that are often isointense with bone in CT images and thus contaminate segmentations that use standard histogram based methods. However the SWD is first performing a quantitative estimate of *the entire volumetric shape* of these structures by fitting them to a set of 3D functions (as described in the “Methods” section), then using the estimated coefficients to detect boundary regions between components (i.e., rock and bone). The results (e.g. Fig. 7B) are thus distinct (i.e., segmented) 3D shapes fully numerically characterized by a set of coefficients that can be used for geometric analysis—computation of volumes, surface areas, etc, and can be compared with other shapes. It should be noted that while bird and reptile brains are smooth, some mammals, especially humans, cetaceans, proboscideans, and artiodactyls are highly folded, and the SWD can be used to quantify the degree of foliation. This analysis has been shown to be useful in the study of cerebellar foliations in elasmobranch brains, which is related to their evolutionary development, predation strategies, habitat, behavior, and cognitive capabilities^51^.

## Discussion

### Volumetric versus surface based methods

The *spherical wave decomposition* (SWD)^52^ was developed to overcome the limitations of surface-based methods by directly analyzing the entire data volume, obviating the segmentation, inflation, and surface fitting steps of surface based methods, significantly reducing the computational time and eliminating topological errors while providing a more detailed quantitative description based upon a more complete theoretical framework for volumetric data. One of its most important features is the lack of topological defects that plague surfaced based methods^52^.

Characterization of morphological features embedded within noisy volumetric data is the essential technical issue in quantitative descriptions of morphology, and so plays a critical role in the characterization of morphology both within and among species. Standard approaches to this problem employ surface based methods. Those that attempt to fit functional coefficients require an initial segmentation of a surface and often a subsequent inflation of this surface to satisfy the uniqueness or stability of subsequent surface fitting algorithms. These methods are inefficient and time consuming because of the need for segmentation prior to fitting and the computationally intensive inflation process, the latter being also a significant source of errors due to creation of topological defects.

It is also worth noting that most implementations of surface-based methods are of the external surfaces, not the internal surfaces, so that a significant portion of the morphology remains uncharacterized. Exceptions to this are surface renderings of internal negative spaces within skulls, such the brain and inner ear spaces and the nasal cavity^49^. However, this type of skull morphometric analyses to use internal anatomy along with external anatomy is uncommon. The SAPID approach presented here makes such analysis straightforward.

Another important feature of our SWD numerical implementation is its speed: it can reconstruct a volumetric data of size $256 \times 256 \times 128$ in the order of about 10 s, as compared to a surface only reconstruction using a standard package used in medical imaging^53^ on the same data, which took $\approx 14$ h. This speed opens up the possibility of automatically exploring very large databases in a practical amount of time, enabling broader intra- and inter-species comparisons.

It should be noted that the volumetric approach emphasized here does not preclude the use in cases where only surfaces, or other substructures, are of interest (and other features ignored), as these are straightforward to extract following the volumetric processing.

### Scientific use cases: pivotal questions

The dominant patterns and processes of morphological change across animal lineages remain subjects of active debate. Morphological change can be modeled as primarily gradual in pattern or as saltatory and punctuated, with rapid accumulations of evolutionary change after long periods of stasis^54^. The processes of morphological diversification among lineages are also contentious^55^. Diversification may occur primarily through widespread adaptive evolution, bringing lineages closer to theoretical ecological or engineering optima. If evolution is primarily driven by different selective pressures then frequent convergence in shape among distantly related species may be observed^56^. Proposed examples of functional-adaptive diversity and convergence include a few different dietary ecologies in hundreds of species in bats^57^, similarities in the dental morphology of mammals adapted to similar diets but representing widely divergent clades^58^; similarities in the distal limb morphology of eutherian and metatherian large mammal predators^59^; and even relatively specialized structures like the “plagiaulacoid” form of premolar seen in multituberculates, marsupials, and plesiadapiforms^60^. Alternatively, diversification in anatomical form may occur primarily along pathways regulated by legacies of constraint^61^. Under this model of diversification, a more limited role of adaptive evolution as well as an extensive role for non-selective drift and historical constraints is acknowledged. This model of limited adaptation within the parameters of constrained inherited body plans was proposed^62^ to describe the evolution of animal life subsequent to the Cambrian. However, as Gould noted, this hypothesis is impossible to conclusively assess in the absence of quantitative methods for capturing organismal shape in the absence of homology. In other words, a key premise of the hypothesis is that differences between phyla should be larger and more environmentally correlated than differences within phyla. But since all shape comparison methods reference specific homologous structures we cannot yet actually comment on whether differences between worms and vertebrates are in fact bigger or smaller than differences within vertebrates.

One might think that finding the answer to questions about evolutionary process is now the purview of genomics since anatomy has a genetic basis and population genetics provides researchers with straightforward tools for determining whether selection has operated or is operating on a given genetic region or locus^63^. However, the ability of genetics to describe the selective basis of differences in complex structural anatomical traits may in fact be quite limited^64,65^. There is growing recognition that patterns of anatomical variation themselves may still hold the most promise, especially when quantified correctly and analyzed in the appropriate statistical frameworks^66–68^. A starting point for such analyses is recognizing cohesive species/population units in the fossil record and extant museum collections, which allows for documenting magnitudes and patterns of intraspecific variation and trait covariance^69–71^. Broadly characterizing general properties of shape variation, while critical for understanding macroevolutionary processes, has stymied evolutionary biologists and paleontologists alike. While there have been a growing number of 3D morphometric studies based on landmark datasets, these are still based on surface based methods, and only register a limited number of preselected landmark points, rather than the entire volume. With the SAPID approach to shape characterization and comparison, not only can we statistically quantify the shape in populations (such as the mean, which provides a template or atlas, and the variance which is the necessary unit of evolutionary change), but we can define shape difference without reference to subject landmarks, linear measures, or ordinations of such subjective variables. In other words, we can define metrics representing variation in overall anatomy without being limited by the presence/absence of particular features, while still quantifying the subregion variation contributing to these overall metrics.

The efficiency and automation of the SAPID algorithms will facilitate exploration of large datasets to investigate the role of natural selection in driving extant vertebrate diversity. Distinguishing epigenetic relationships from change driven by natural selection is a tenacious problem. This is currently not within the capability of existing algorithms. For example, with large virtual collections of specimens as CT data, one could investigate the hypothesis that natural selection has had a significant and primary effect on cranial and skeletal anatomy. The scan data combined with SAPID methods would allow one to evaluate the prediction that (a) those groups with greater ecological diversity should have greater cranial and skeletal diversity when estimated age of the group’s common ancestor is taken into account, (b) and that when grouped by ecological niche, different clades of primates or classes of vertebrates should show common trends relating and distinguishing those niche groups.

### Development of standardized analysis and reproducibility pathways

SAPID demonstrably represents a fundamental advance in methods for volumetric shape comparison and image registration from a theoretical, mathematical, and computational perspective. However, it is not necessarily intuitive to most morphometricians for some of the same reasons. The most fundamental difference between SAPID and traditional shape comparisons is that ‘shape distance’ between two objects is not uniquely defined as it would be in comparing landmark datasets in geometric morphometrics via Procrustes distances. Instead, with SAPID, it is possible to control the ‘goodness’ of fit between two distinct shapes by modifying parameters used to generate the symplectomorphic mapping, thus producing results with different “shape distances” for the same two shapes. This could be frustrating for morphometricians because it superficially adds a new level of ambiguity to image comparison. However, such ambiguity, in truth, has always been there. In traditional geometric morphometrics, it is masked by the problematic assumption that affine transformations are biologically and geometrically sufficient, and that a researcher’s particular choice of landmarks and the between-species deformations implied by those landmarks are meaningful descriptions of the geometry. Allowing the first of these assumptions to monopolize geometric morphometrics risks misinterpreting the repeated observation of patterns due to this approach as fundamental patterns of shape variation, which could mislead the development of broad evolutionary theories. The second set potentially adds to problems of reproducibility between researchers and fundamentally prohibits comparing variation among structures that are not homologous on some level.

A non-unique distance measure leaves two possible routes for assessing degree difference between morphological samples. (1) Use a biological control (aka reference) sample to define the appropriate parameters of image registration, and apply these to experimental perturbations of the sample or comparisons with other samples. (2) Use a critical measure of shape difference (e.g., zero difference), to determine the appropriate parameters for image registration between any two objects. In this second scenario, understanding and quantifying biological variation between samples will require introduction of some kind of registration metric that can possibly incorporate the magnitude of the deformation and/or the parameters of the fit needed to perfectly match individuals to each other and/or a template representing the average.

The benefit of the first approach is that it shares more similarity with other current methods of shape comparison and we can already define shape statistics for these comparisons (e.g. parameters of image registration can even be chosen to keep the deformations uniform across the volume or across each coordinate independently, thus resulting in familiar orthogonal or affine transforms)^31^. The drawback is that it requires the assumption that particular control samples have been appropriately defined, it requires the preservation of these control samples for reproducibility, and it means that conclusions of studies hinge on the composition of their control samples. A further problem with this approach is that, while it should usually produce satisfying results in suggesting large quantitative differences among qualitatively different shapes, the registration of these shapes often will not make much biological sense to researchers, leading them to question the utility of the distance.

The benefit of the second approach is that forcing a complete registration between two objects leaves little ambiguity about the correctness of the mapping (researchers can easily verify that obviously homologous structures (e.g., nasal apertures, orbits, neurocrania) are mapped to each other. The drawback is that statistics for parameters describing the amount deformation used to accomplish the registration are not yet defined. Defining statistics for this mode of comparison is thus an area of important work.

Finally, one opportunity presented by the SAPID approach to shape registration is that effects of non-linear differences in position of homologous objects (like differences in postures of two articulated skeletons) can be separated from differences due to registering their volumes to one another. If implemented into an analytical workflow, scans of multiple skeletons of the same species could be retro-deformed into similar postures and then compared for whole skeleton morphological variation.

Another area of future work is the assessment of sensitivity to control samples and problems with mappings of qualitative different shapes. Possible approaches to this problem include (1) iteratively permuting composition of control samples for various bones of various species to assess the sensitivity of intraspecific mappings to these variables. While helping us understand the effect of robustness of intraspecifically (or subspecifically) defined parameters, this will allow comparison of heterogeneity in variation among species and among anatomical regions which will also address interesting biological questions.

The SAPID analysis can be used to augment existing taxonomic strategies. For example, after computing within and between species differences using parameters based on one or several intraspecific samples, the matrix of interspecific differences can be used to define the minimum spanning tree relating those samples where the length of a connection between species, which is defined as the shape differences between two species templates under the predefined image registration parameters. This graph can be used to compose (or guide) mappings between species with large qualitative differences thereby avoiding distances based on unlikely registrations but maintaining a constant set of registration parameters.

### Hypothesis testing

In the end, what is of greatest importance for morphometric methods is their utility in hypothesis testing. This requires the ability to quantitatively compare complex shape variations within or between species over time. The more precise the geometric characterization of a volumetric shape and the registration between specimens, the greater the sensitivity to subtle variations hypothesized in evolutionary models. This is ultimately the greatest power of the SAPID method, which provides such precision in the very general context of volumetric imaging data, be it from MRI, CT, or any other scanner type.

A specific example that might be helpful in clarifying this point is provided by the question of the coevolution of the mammalian middle ear and neocortex examined in^44^ using CT data of *Monodelphis domestica*. At the time this paper was written (${\sim }\, 25$ y ago), 3D analysis methods were far less developed. Studying the development and growth of the didelphid mandibular arch was done by examining how CMJ and middle ear growth relate to each other in the context of cortical plane growth. The CMJ is a fixed reference point to align the different individuals to show the growth trajectory of the fenestra vestibuli, and outlines of the ’cortical equator’ were used to represent brain growth. This required manual tracing of structures in a single slice, identifying appropriate landmarks for comparison, then merging the results onto a single image (see Fig. 1d in^44^). This took several weeks of work to complete.

That same CT data is shown Fig. 1(left column) where the entire dataset, or any substructure(s) of interest, can be quantitatively characterized and compared between species and over time. In Fig. 1(middle column) we have demonstrated the simplest form of such a comparison—the decreasing mean squared difference in shape between maturing specimens and a reference adult specimen, just to emphasize these capabilities. In the practical application to this problem, SAPID could be used to examine the particular substructures of interest (the CMJ and fenestra vestibuli) and provide quantitative characterizations of the shape variations in terms of whatever metrics are deemed of greatest interest, such as distances, angles, mid-points, shape complexity (i.e., number of SWD coefficients necessary to characterize the shape, etc). These would provide more precise quantitative measures that can then be used in a hypothesis-driven analyses of shape association with particular factors such as diet/locomotion, disparity measurements, and sometimes estimates of integration and/or modularity—more often than not in a comparative/phylogenetic context. Such work is beyond the scope of the current paper but will be addressed in a future study.

It is important to recognize that the SAPID methods actually broaden the scope of hypothesis driven research. The current standard methods for shape comparison that involve identification and subsequent registration of landmarks identify these landmarks a priori, and thus there is an implicit bias as to their relevance. On the contrary, SYMREG will automatically register the entire shapes. Standard landmarks (homologous structures) will automatically be registered but any additional co-registered shapes can be identified ex post facto as useful landmarks, potentially providing new hypotheses about the evolutionary trajectory. These methods might usefully inform more current research on a wide variety of topics (e.g.,^72–74^).

The important overarching point is that the ability of the SAPID approach to produce quantitative volumetric characterization and registration provides the basis for utilizing a wide range of standard statistical comparison methods with much greater sensitivity to geometric variations in specific tissue types than extant methods. This can result in an enhanced ability to more precisely quantitatively address significant specific questions in evolutionary biology.

## Conclusion

In conclusion, we have presented two theoretical constructs and their numerical implementations, the *spherical wave decomposition* (SWD)^42^ that provides fast, accurate automated characterization of shapes embedded within complex 3D datasets, and *symplectomorphic registration with phase space regularization by ESP* (SYMREG)^43^, a volumetric non-linear registration method that allows homologous structures to be correctly warped to each other or a common template for comparison. Take together, these constitute an automated approach to true volumetric computational morphometrics that we call the *Shape Analysis for Phenomics from Imaging Data* (SAPID) method. This paper has shown its capability on several datasets of importance to evolutionary biology, paleobiology, and digital library data usage, which suggests its widespread utility in a broad scope of these and related disciplines.

## Methods

### Volumetric shape analysis

The goal of shape analysis is to quantitatively characterize a 3D “object”, such as a skull specimen, that has been imaged by some volumetric imaging modality, such as computed tomography (CT). Specifically, the objective is to represent the digitized version of the object by some well-defined mathematical functions to assign numbers containing the shape information that uniquely describe the specimen and can be used to compare to other specimens. It is useful to think of this process as involving two steps: (1) Choosing a set of mathematical functions appropriate to generally describe such objects, which we will call the issue *representation* and (2) Determining the parameters of that representation that uniquely describe a specific volumetric image of a specimen, a process called *reconstruction*.

The goal of quantitative morphology is to construct a numerical characterization of the spatial organization of an object. There are often many ways to do this but implementing an efficient numerical method can be greatly facilitated by a judicious choice of the representation. One way to think about this problem is as a decision about the choice of coordinate system. This is best illustrated by three examples, which will quickly take us to the motivation for the SWD.

#### Cartesian basis and spatial coordinates

Consider the problem of analyzing very regular shapes, such as an office building which we assume to be a perfectly rectangular cuboid. The shapes are characterized by the three numbers—the length *a* along one base, which will call the *x*-axis, the length *b* along the other base, which will call the *y*-axis, and the height *c* along the vertical or *z*-axis. Since these directions are all perpendicular, we can define a vector of unit length along each direction:

1 $$\begin{aligned} \varvec{e}_{x} = \begin{pmatrix} 1 \\ 0 \\ 0 \end{pmatrix}, \quad \varvec{e}_{y} = \begin{pmatrix} 0 \\ 1 \\ 0 \end{pmatrix}, \quad \varvec{e}_{z} = \begin{pmatrix} 0 \\ 0 \\ 1 \end{pmatrix} \end{aligned}$$

and then any point *f*(*x*, *y*, *z*) within the building can be described by

2 $$\begin{aligned} q(x,y,z) = \alpha a \varvec{e}_{x} + \beta b \varvec{e}_{y} + \gamma c \varvec{e}_{z} \end{aligned}$$

where $0 \le \alpha ,\beta ,\gamma \le 1$. The vectors in Eq. (1) are called *basis* vectors of the Cartesian coordinate system. The construction in Eq. (2) is called a *linear combination* of basis vectors and it works because the basis vectors are mutually perpendicular, or *orthogonal*. This important property will be shared by the other bases we use below. A basis can be thought of a set of functions that can completely and uniquely describe all points in a space. In order to strictly qualify as a basis, certain properties are required, which go beyond the scope of this paper but can be found in any linear algebra book^75^.

Characterizing the shape of the building—its volume, distance between the ground floor entrance and your desk on the 10th floor, the volume of a substructure (such as a floor) can all be done using these basis vectors. We just use points along these axes and the rule for vector addition and we can compute the geometric quantities of interest—distance, area, volume, etc.

#### The frequency domain and plane waves

Unlike an office building, biological objects such as the CT of a skull or the MRI of a brain have exceedingly complex geometries, so a more general approach is necessary. One feature of biological structures that can be used to advantage is that they tend to exhibit extended spatial patterns, though these maybe complicated. This suggested working in a coordinate system of patterns, rather than directly in the spatial coordinates. A simple example is the very regular pattern of a sine wave in space, which in one-dimension is:

3 $$\begin{aligned} s(x) = A \sin (k x + \theta ) \end{aligned}$$

where *x* is the spatial coordinate. This wave is described by only three parameters: (1) its spatial frequency in the *x*-direction *k* (in units of $1/\text{distance }$)—how rapidly it oscillates as a function of position; (2) its amplitude *A*—the height of the peak; and (3) the phase $\theta$—the offset of the starting point. In other words, the best coordinate system to describe such a wave is *not* the spatial domain $\{ x,y,z \}$, but rather the coordinates $\{ \omega ,A,\theta \}$. This is called the *spatial frequency domain*. The variables *x* and *k* are called *conjugate variables* because we can characterize the wave equation (3) in either domain.

Somewhat surprisingly, more complex shapes can be described by the combination (i.e. the sum) of waves of different amplitudes, spatial frequencies, and phases. This is the essence of *Fourier’s Theorem*. The remarkable property of the $\sin$ and $\cos$ functions is that they are mutually orthogonal and form a basis in frequency space, in the same way that the vectors equation (1) formed a basis in coordinate space. Frequency space is also called *Fourier space* and the trigonometic functions are called the *Fourier basis functions*.

Recalling that $\sin$ and $\cos$ are just phase shifted versions of one another, we can eliminate the explicit phase $\theta$ and write such a combination of waves in one-dimension at a single point $x_{i}$

4 $$\begin{aligned} s(x_{i}) = \sum _{j=1}^{m} \left( a_{j} \cos k_{j} x_{i} + b_{j} \sin k_{j} x_{i} \right) = \sum _{j=1}^{m} c_{j} e^{i k_{j} x_{i} } \end{aligned}$$

where *m* is the number of spatial frequencies in the shape and the phase $\theta$ is now contained in the relative amplitudes of *a* and *b*. The last equality is a consequence of Euler’s relation $e^{i b} = \cos b + i \sin b$ where $i = \sqrt{-1}$ defines a *complex number* which can be thought of as a convenient bookkeeping device that makes the equations much easier to work with as it avoids a proliferations of terms.

This notation is particularly convenient because we can easily generalize the expression for the signal at any point $\varvec{x}_{i}$ to 3-dimensions as

5 $$\begin{aligned} s(\varvec{x}_{i}) = \sum _{j=1}^{m} c_{j} e^{i \varvec{k}_{j} \cdot \varvec{x}_{i} } \end{aligned}$$

where bold-faced represents vectors, which in this case are $\varvec{x} = \left( x,y,z \right)$ (a standard, if somewhat confusing reuse of *x*) and $\varvec{k} = \left( k_{x},k_{y},k_{z} \right)$, and the dot represents the dot product, i.e. $\varvec{k} \cdot \varvec{x}_{i} = k_{x} x_{i} + k_{y} y_{i} + k_{z} z_{i}$. In this representation the waves are perpendicular to planes in 3D space and so are called *plane waves*. Equation (5) is called the Fourier Transform of the coefficients *c*. It converts the coefficients in the frequency domain into a signal in the spatial domain. Using Eq. (5) we can write the expression for data collected at *n* points $\varvec{x} = \{ x_{1}, \ldots , x_{n} \}$ represented by *m* plane waves in compact matrix form as

6 $$\begin{aligned} s(\varvec{x})&= \varvec{F} \varvec{c} \nonumber \\ \text{ where } \quad \varvec{F}&= \{ e^{i \varvec{k}_{1} \cdot \varvec{x}}, \ldots , e^{i \varvec{k}_{m} \cdot \varvec{x}} \} \nonumber \\ \varvec{c}&= \{ c_{1}, \ldots , c_{m} \} \end{aligned}$$

where $\varvec{F}$ is a matrix if size $m \times n$ and $\varvec{c}$ is a vector *m*. This form will prove useful when we turn to the problem of reconstruction.

**Figure 8:**
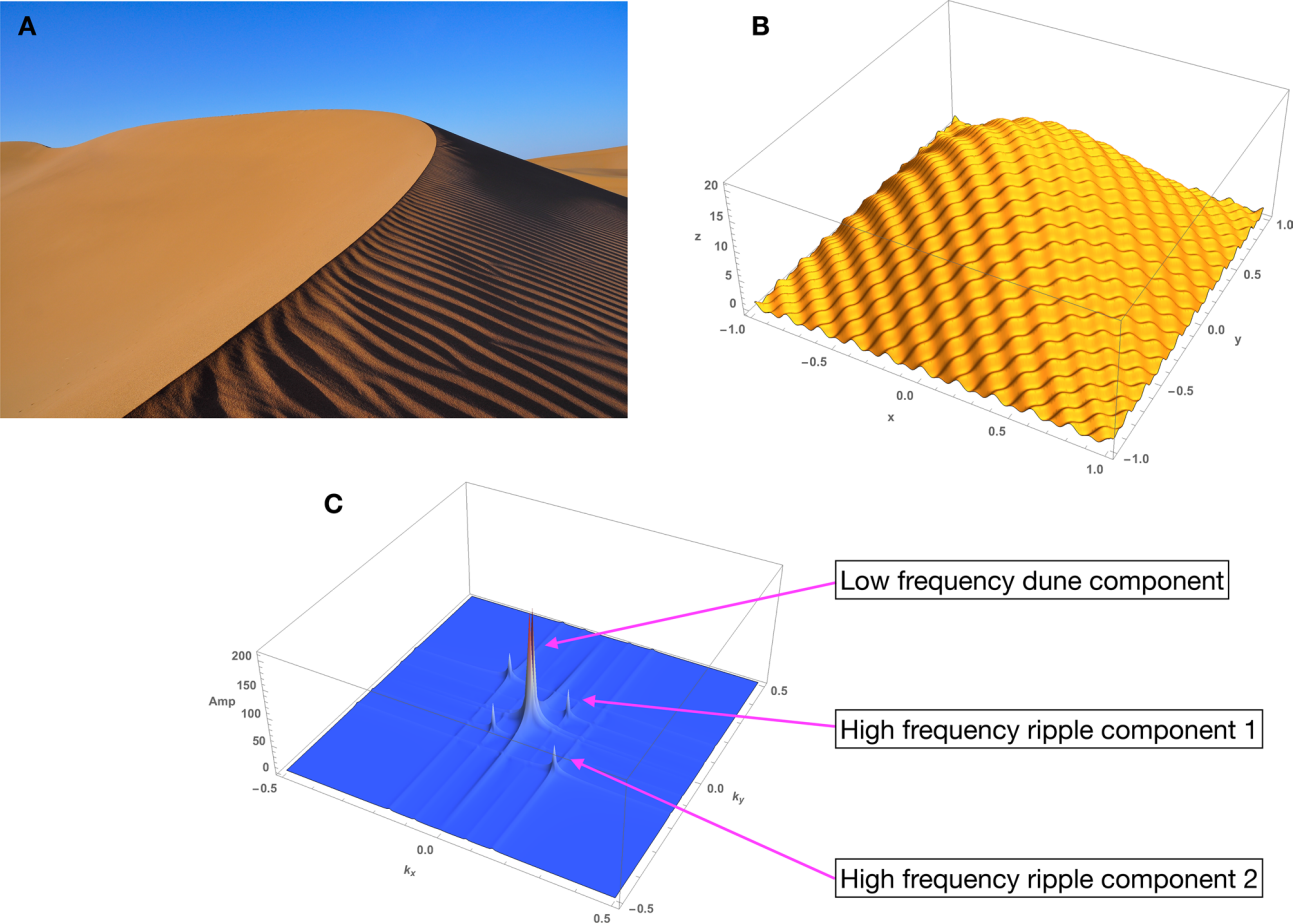
Shape characterization with Fourier plane waves. (**A**) Real sand dune, with surface ripples. (**B**) Numerically generated (idealized) sand dune with ripples. The underlying dune is represented by a single low spatial frequency, high amplitude wave. The ripples were generated by the sum of two high frequency, low amplitude waves oriented at $45^{\circ }$ and $-45^{\circ }$ relative to the *y*-axis. The entire dune is the sum of all these waves. (**C**) The magnitude of the Fourier Transform (FT) of (**B**) detects these three waves. The location of the peaks gives the spatial frequency, the angle of the peaks in the $(k_{x},k_{y})$ plane provides the angle in the spatial (*x*, *y*) domain. These estimated Fourier components characterize the dune shape. The mirror image peaks are redundant information that result from the symmetry of the FT. The small ridges emanating from the peaks are a consequence of the finite nature of the numerical FT.

A simple example of the Cartesian Fourier (i.e. plane wave) representation is the description of the shape of a sand dune that has wind-blown ripples on its surface, as in Fig. 8A. A highly idealized mathematical representation, for illustrative purposes, is shown in Fig. 8B. The underlying dune is constructed from a low spatial frequency but high amplitude wave and the ripples are created from a high spatial frequency but low amplitude wave. The entire dune is the sum of these waves.

The representation of a *generic* object in terms of a set of basis functions can be thought of as the construction of a *model* for the data. Characterization of a *particular* object requires finding the parameters of that model that produces the most faithful representation of the object. This is the problem of *reconstruction*. It is an *inverse problem* that requires methods of inference, and hence probability theory. The example of Fourier reconstruction provides a nice example of the power of probability in reconstruction problems (see, for example,^76^) but is beyond the scope of the present paper.

From Eq. (6), given the data $s(\varvec{x})$ the estimated coefficients $\hat{\varvec{c}}$ can naively be estimated by using the Inverse Fourier Transform

7 $$\begin{aligned} \hat{\varvec{c}} \approx \varvec{F}^{\dagger } s(\varvec{x}) \end{aligned}$$

where $\varvec{F}^{\dagger }$ is the complex conjugate transpose of the model functions. The result of this procedure for the sand dunes is shown in Fig. 8C, which shows that the shape, characterized by the Fourier *spectrum* is reasonably retrieved. It is important to recognize, however, that Eq. (7) is formally *not* the correct answer, even though in many cases it is a reasonable approximation. *But generally reconstruction is not the same thing as multiplying by the inverse of the basis functions!*. For example, in standard least-squares estimation, **c** would be reconstructed by replacing ***F***^†^ with the pseudo-inverse of ***F*** in Eq. (7). This example was chosen for its simplicity and because the constituent waves are visible as such. But much more complex geometries that do not appear wavelike can be constructed by the same approach, as we shall see.

#### Spherical basis and spherical waves

In the sand dune example we ignored the fact that the sand dune is sitting on a curved surface—the Earth. This was reasonable because the curvature of the Earth is insignificant over the extent of the dune so that, for all intents and purposes, the dune is sitting on a flat surface. Mathematically, we say that the Earth is *locally flat* in the region of the dune. This problem is therefore naturally described as Fourier functions in Cartesian coordinates—just the plane waves discussed in the previous section.

**Figure 9:**
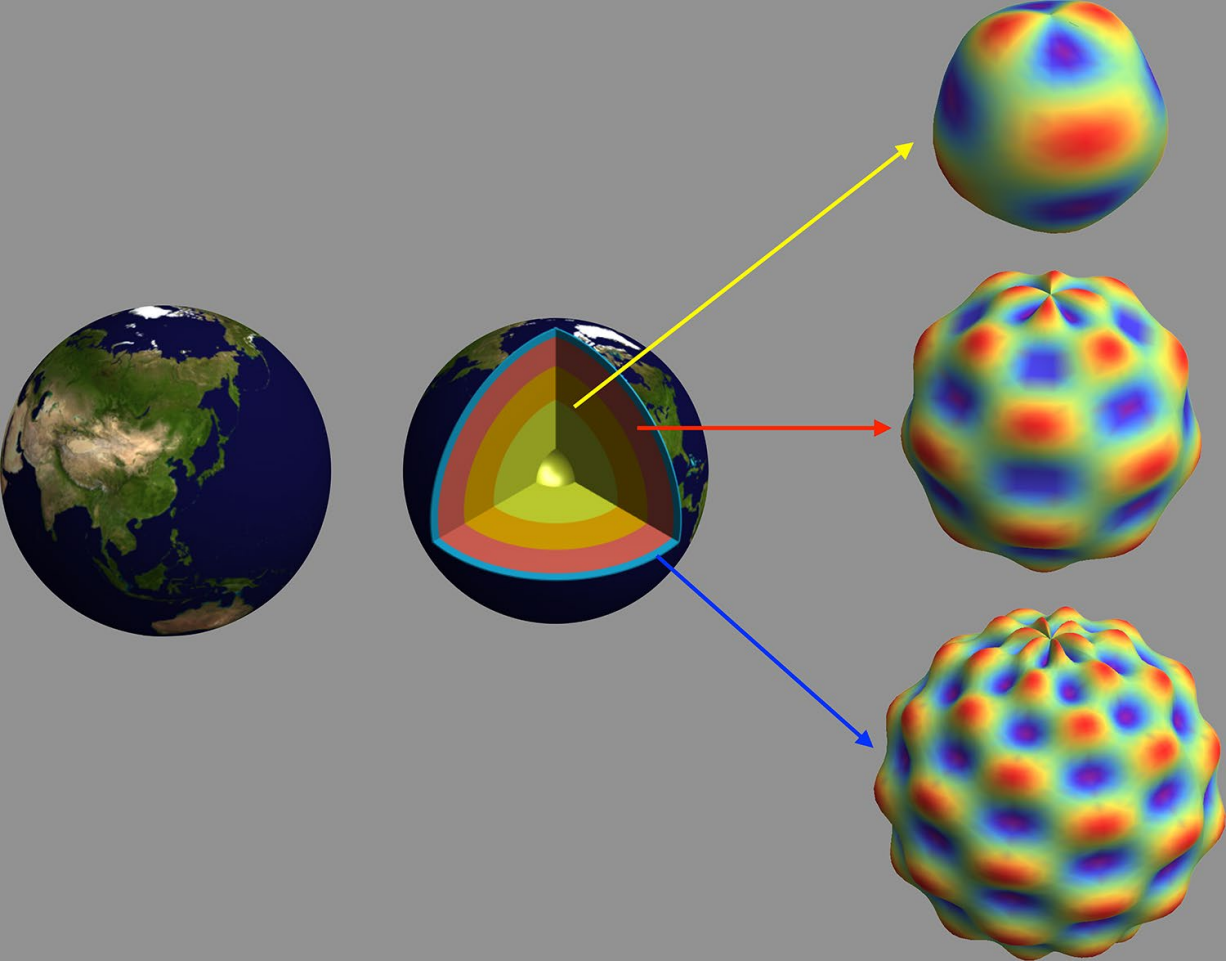
Shape characterization with Spherical Waves. (Left) Schematic of the geological layers of the Earth, each of which is idealized to have a mean spherical shape but complicated surface features (e.g., mountains and valleys on the outer, or crust, layer). (Right) Schematic of spherical waves fit to each layer consisting of variations in both the radial and angular dimensions. No data was actually fit, and the features size is greatly exaggerated for emphasis. In this example the complexity of the surface is presumed to increase with radius just for illustrative purposes. Only the surfaces of each layer has been “fit” for clarity but in practice a continuum of fits over all radial values is performed.

But what if we wanted to characterize the entire surface of the Earth, not just a single dune? And then go even further and examine the surfaces of the major interior layers of the Earth (Fig. 9)? For this there is a more efficient, and intuitively obvious, coordinate system called the *spherical coordinate system*, parameterized by the three coordinates $\{ r,\theta ,\phi \}$ which are the *radius* *r*—the distance from the center of a sphere to a point on its surface, such as the top of a mountain, the *polar angle *$\theta$—the angle from the geographical (not magnetic) North pole, and the *azimuthal angle* $\phi$—the angle along the Equator, measured from some reference point (say, the Greenwich Meridian). These are more familiar, of course, as *altitude*, the *latitude* and the *longitude*. (Actually the latitute is measured from the equator, so $90^{\circ } - \theta$, but the lines of constant latitude are the same).

The spherical coordinate system does not provide any more information than the Cartesian coordinate system (indeed, one can transform from one to the other), but it is more natural, and provides more parsimonious descriptions. For example, the path of someone traveling on an arbitrary path on the surface of a sphere can be specified by only two parameter—the angles $\{ \theta ,\phi \}$, since the radius *r* is a constant. In Cartesian coordinates, description of the same path would require all three parameters $\{ x,y,z \}$.

Let’s consider first a hypothetical situation in which the Earth’s surface was entirely covered by giant sand dunes. And for simplicity let’s assume that the shape of the Earth underneath the dunes is a perfect sphere (it’s actual an oblate spheroid). How do we describe the wave in spherical coordinates so as to take into account the curved surfaces on which the waves are propagating? Well, it turns out that the Fourier plane wave functions developed in a Cartesian coordinate system can be rewritten in spherical coordinates using special functions: *spherical harmonics* and *spherical Bessel functions*. The combination of spherical harmonics (the angular variations) and spherical Bessel functions (the radial variations) are called *spherical waves*. These are the functions that we will use to characterize the entire shape of the digitized 3D objects within the volumetric images.

The characterization of the Earth’s volumetric features in terms of spherical waves is shown schematically in Fig. 9. No actual fitting has been performed, and the surface features are idealized and exaggerated—this is just an illustration of the ability of spherical waves to characterize both radial and angular variations volumetric shapes.

**Figure 10:**
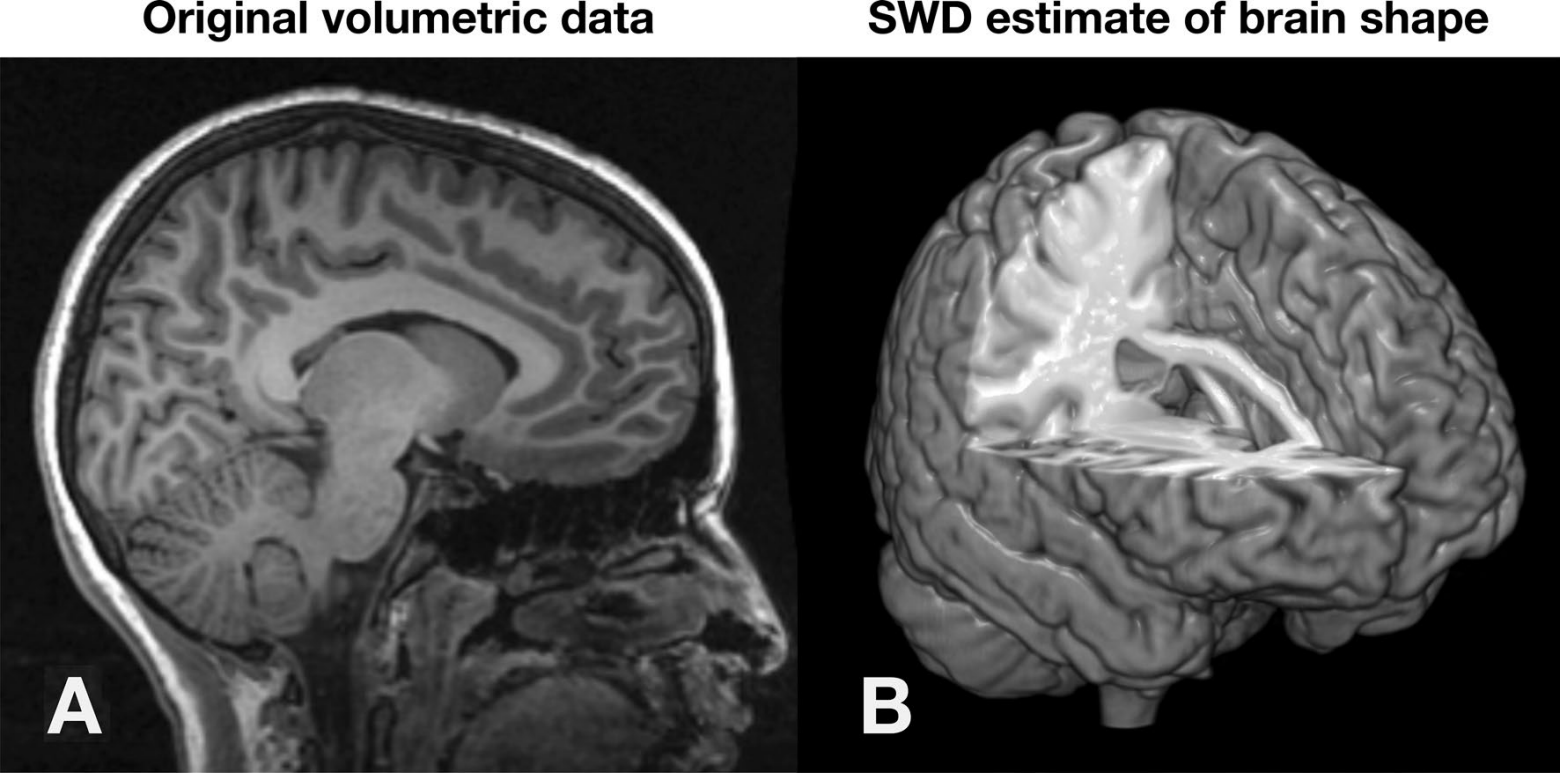
Automated human brain shape characterization by Spherical Wave Decomposition (SWD) from a high resolution anatomical MRI scan. (**A**) Original volumetric MRI scan, (**B**) SWD estimate of the brain shape (adapted from^42^). The shape in (**B**) is characterized by the specific set of $N=100$ SWD coefficients, as well as by the number *N* itself, which is calculated as the “optimal” number of coefficients and is thus a measure of the brain shape complexity (see^51^).

Determining the coefficients that characterize the shape of the object through these spherical wave functions is what we call the *spherical wave decomposition*^42^. An example of volumetric shape characterization of a single high resolution anatomical MRI brain scan is shown in Fig. 10.

#### The model order problem

There is an insidious problem lurking in the previous examples. In Eq. (5) the label *m* represents the number of model functions necessary to completely described the shape. This is called the *model order*. But how many basis functions do we need to accurately and optimally characterize the shape or, alternatively, what is the *optimal* model order $\hat{m}$? In the sand dune example, this problem was hidden because we constructed such a simple model with separate spatial frequencies that were well resolved. And there was no noise. But with noisy data that contain a wide spectrum of spatial frequencies, we will no longer be able to ignore this issue.

**Figure 11:**
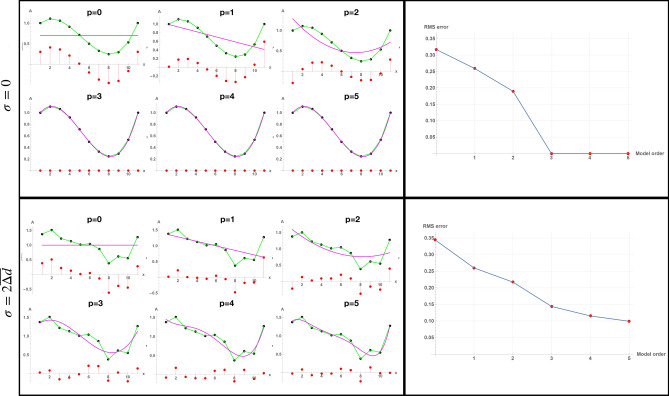
The model order problem. In the left column is shown the “data”, which consist of a third-order polynomial (green). The shape estimated from a standard least-squares fit is shown in magenta for hypothesized model order *p*. Deviations between the fit and the data at each point are shown as red dots. In the right column is shown the RMS error between the estimated shape and the data as a function of model order. In the **top row** there is no noise ($\sigma = 0$) and the shape is faithfully reconstructed (i.e., $RMS \approx 0$) at the correct model order $p=3$. Subsequent fits at higher order $p>3$ also provide correct fits, but are no improvement over the correct solution of $p=3$, as seen by the RMS vs model order plot on the right. The “optimal” fit order is thus estimated to be where there is no RMS improvement, which is $p=3$. In the **bottom row** the same analysis is performed but the data is now contaminated with random zero-mean Gaussian noise with $\sigma = 2 \overline{\Delta d}$ (twice the mean deviation of the data). The RMS versus model order plot on the right no longer shows a sharp distinction at the true value of $p=3$ and continue to decrease as the higher order components of the shape *due to the noise* are increasingly well fit.

**Figure 12:**
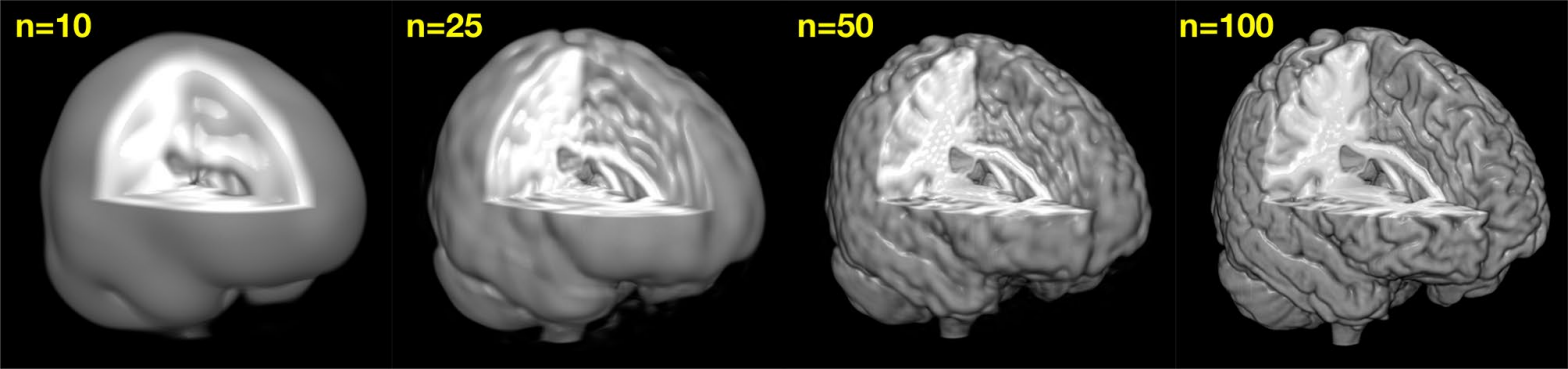
The model order problem in volumetric SWD analysis. The optimal fit shown in Fig. 10B was determined by searching through a range of model orders *n* (shown in yellow) and determining statistically which value first reduced the mean squared error to a minimum to within the noise (the equivalent of $p=3$ in Fig. 11). See^42^ for details.

Every imaging process has limited resolution because of sampling restrictions and is contaminated by imperfections that we will generically call “noise”. In addition, the number of functions necessary to characterize the shape, also called the *model order*, is another unknown. The goal of the reconstruction process is to determine the coefficients of the data model and the correct model order from the noisy volumetric data of finite resolution. We can illustrate the problem with a very simple example of fitting a polynomial discussed in Fig. 11. An example of this applied to a volumetric human brain image using the SWD is shown in Fig. 12.

The model order has physical significance as a measure of an objects complexity, and consequently can have biological and evolutionary significance, as was demonstrated in our study to characterize of cerebellar foliations in elasmobranch brains^51^, which has implications in the quantification and comparison of the cerebellum in different species of elasmobranchs where variation in cerebellar foliations has been shown to be related to species habitat and predation strategies and has evolutionary significance^77–79^.

#### Volumetric segmentation

One important difference between the SWD and surface based methods is that the SWD provides a method for naturally and effectively performing the very complex task of volume segmentation. Segmentation is not tractable by surface based methods alone because they are themselves predicated on segmentation. That is, segmentation must be performed (either by hand or by using additional specialized semi-automated segmentation tools) *before* the surface based methods can even be applied to new volumetric data. In the SWD approach, the segmentation is done *on the entire volume*. All important features of the SWD approach (including weighted Fourier smoothing, optimal SWD order and volume morphometry/complexity) are also applicable to segmented and independently represented structures.

**Figure 13:**
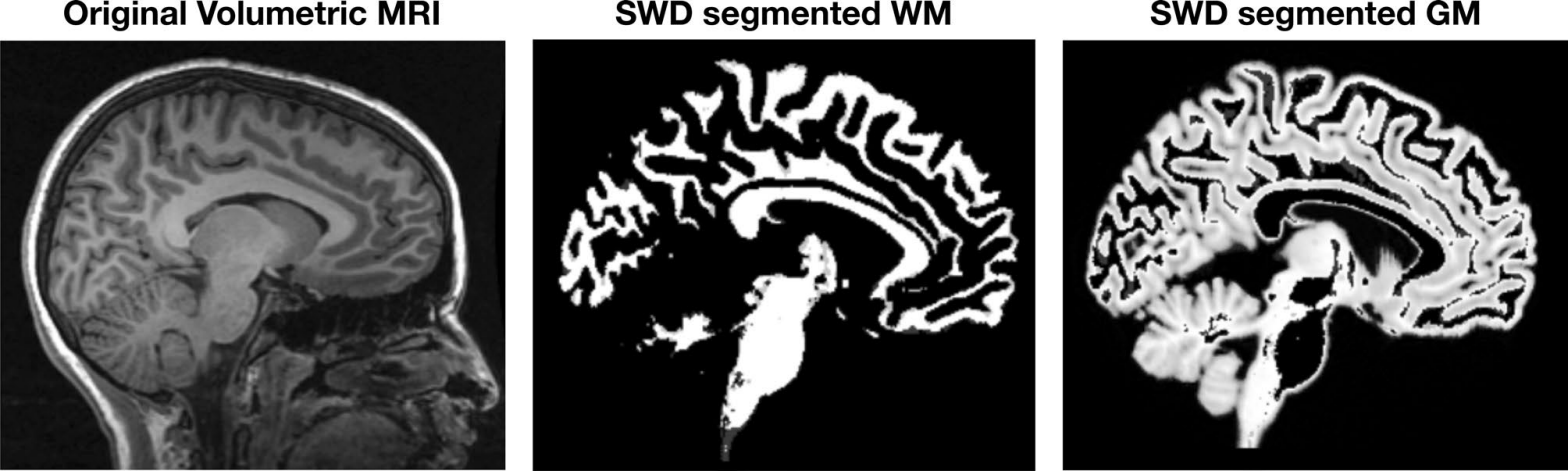
Automated human brain tissue segmentation using the Spherical Wave Decomposition (SWD) on a high resolution anatomical MRI scan. (**A**) Original volumetric MRI scan, (**B**) segmented white matter (WM) and (**C**) gray matter (GM) (adapted from^42^). Each of these segmented tissues are individually described by their own SWD, and thus can be used for quantative comparative analysis.

Because the SWD estimates fit coefficients for a set of volumetric functions, the coefficients for the analytic expansion of the derivatives is produced as well. This automatically provides an estimate of the coefficients of tissue border regions and thus allows automated segmentation of tissue types. This is shown for the human brain data in Fig. 13 where separate volumes for the two tissue types, white matter (WM) and gray matter (GM), are automatically produced.

#### Summary

The SWD produces a quantitative description of an entire volume of data. This output can then be used for comparison between datasets. Comparison of homologous structures between datasets requires a method for non-linear registration, which we discuss next.

### Volumetric image registration

#### The registration problem

The general idea behind geometric morphometrics is to transform different images to the same coordinate system so that relations between homologous points can be determined. Approaches to this problem of registration, or spatial normalization, fall into three basic categories: (1) Rigid registration, which seeks a linear transformation that best aligns two objects without altering the objects’ size and shape, and thus involves only translation and rotation; (2) Affine registration, which seeks a general linear transformation that allows objects’ global size and shape to be altered and thus involves shear and scaling in addition to translation and rotation; and (3) Diffeomorphic registration, a non-linear operation that, in addition to affine registration, normalizes the objects’ size and shapes as well. Not surprisingly, it is also the most complex.

However, current geometric morphometrics^80–83^ is typically based on some form of Generalized Procrustes Alignment (*GPA*)^25,31^ which is based upon a minimum Procrustes distance over all affine transformations^84^ and then performing a subsequent statistical analysis. Unfortunately, the affine methods are only approximations (and sometimes quite poor) for matching, comparing, or combining homologous anatomical structures because affine registration is a global transformation and is incapable of capturing localized changes in shape. In many cases of interest, the localized changes are the most important part. The Procrustes method is rather simplistic in this respect as the geometric differences are essentially distributed as evenly as possible among all landmarks representing each specimen since it aligns specimens by their centroids and then rotates them to minimize the summed distance among all landmark coordinate pairs.

A review^82^ discussing the state of geometric morphometrics states that the 3D extension of 2D methods (namely, Procrustes superimpositions) is straightforward, but the methods discussed only represent affine transformations. These linear methods (utilized in the popular TpsRelW^85^) are currently the standard (e.g.,^39^). Correctly extending non-linear methods to volumetric data is a difficult theoretical and computational problem, encountered often in MRI where it is necessary to compare volumetric data from multiple subjects.

#### The SYMREG method

**Figure 14:**
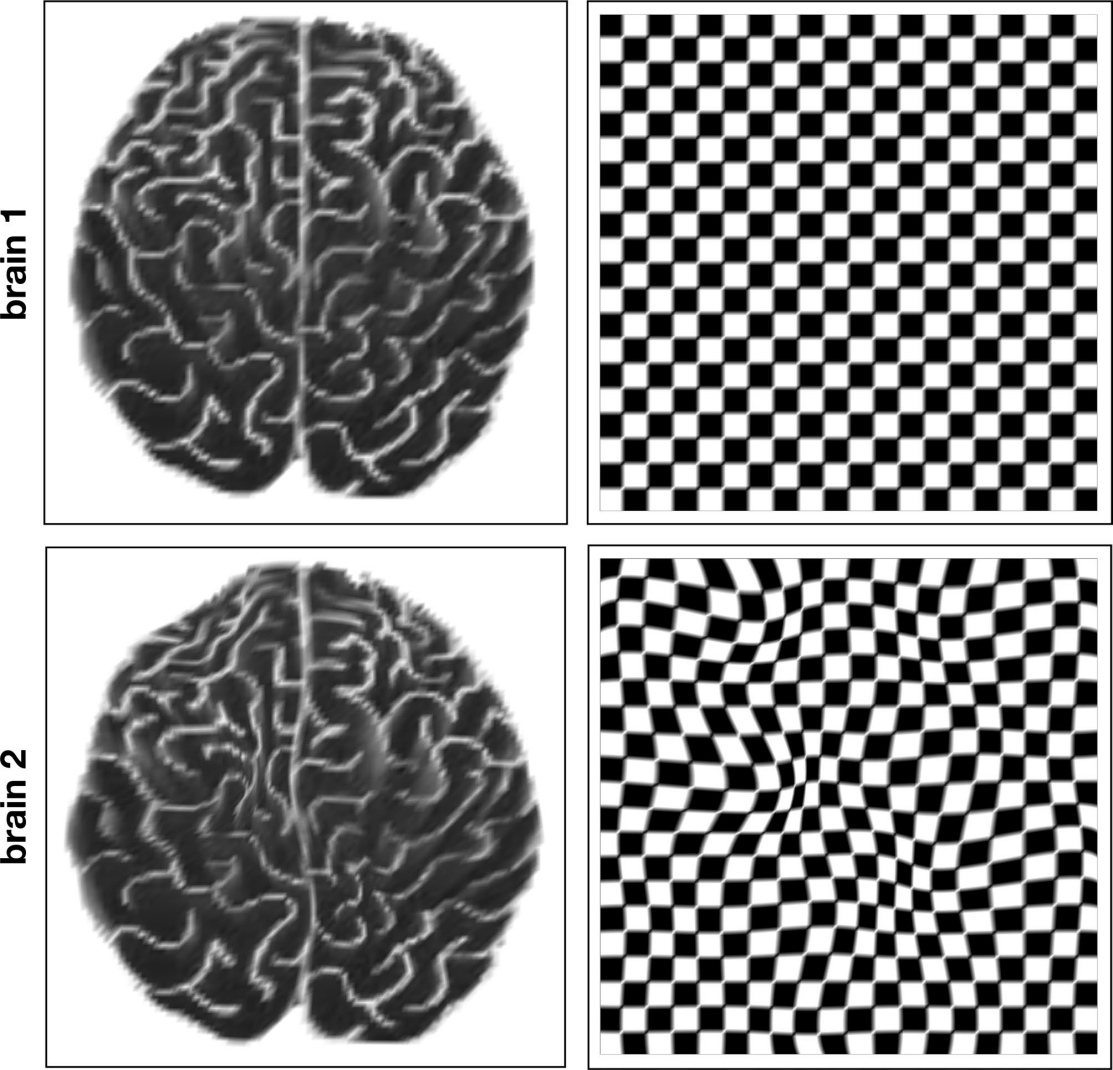
Non-linear variations between brain. While two normal brains have the same structures, the geometrical relationship between them generally varies in a non-linearly fashion. If brain1 (top) is assumed to lie on a perfectly Cartesian grid, the relative geometry of brain2 can be thought of as lying on a nonlinearly distorted version of brain1 grid. SYMREG estimates these distortions and maps brain2 to the grid of brain1. This example is in 2D but SYMREG operates in 3D on the full volume.

The ability to do accurate quantitative geometric morphometrics is predicated on the ability to address the non-linear registration problem. A conceptual way to think about this problem is that two different volumetric data sets have different non-linearly related coordinate systems. The goal of registration is the put all data into the same Cartesian coordinate system. A schematic of this problem is shown in Fig. 14.

As remarked above, diffeomorphic methods are the current standard for current non-linear registration. However, despite their ubiquity, in practice these methods have significant limitations in speed and accuracy that compromise their practical utility^86,87^. The SYMREG method is similar in spirit to diffeomorphic mapping, but is more general and flexible. This development was motivated not only to address the issues of speed and accuracy, but also to facilitate merging of multiple imaging modalities with different resolutions. This has implications for computational morphology where different modalities, in particular both MRI and CT, are in common use.

The SYMREG method is developed within a coordinate space that is more general than just the spatial coordinates used for diffeomorphic methods. Registration methods work by warping the coordinates of one spatial grid onto another. The target grid is called the template (the undistorted Cartesian grid in the top row of Fig. 14). Each spatial coordinate in the warped grid (the grid in the bottom row of Fig. 14) must therefore move along some non-linear path at some rate determined by the multiple steps of the numerical algorithm which iterates towards a final spatial configuration that minimizes the error between the warped grid and the template grid. Therefore each point has a “velocity” in addition to a position. Systems with both a spatial coordinate *q* and a velocity *v* (or more generally, a momentum *p*) can be characterized by the space (*q*, *p*) that contains both coordinates. This is called the *phase space* of the system. The dynamics within this space can be described a function *H*, called the *Hamiltonian*, which can be thought of as characterizing the energy of the system, and can be used to impose constraints that reduce the space of possible solutions, thereby increasing speed and accuracy. Specifically, whereas current methods employ diffeomorphic transformation in spatial coordinates, SYMREG is *diffeomorphic in phase space*, which is called a symplectomorphism.

There is another important and unique aspect of the SYMREG method. Image grids are characterized by the relative spatial locations of the points. Incorporating this *relative* information between points can be used to impose addition constraints that incorporate position dependent information into the problem. A very general and flexible way to do this is using our theory of Entropy Spectrum Pathways (**ESP**)^88^ where coupling between neighboring locations, constructed using a spatially dependent coupling density $Q(\varvec{x},\varvec{x}')$ matrix, results in the ability to constrain, or *regularize*, the solutions with interactions that extend spatially away from the individual points. These are called *non-local* interactions. What this means is easily understood in simplest case that $Q(\varvec{x},\varvec{x}')$ depends only on the different in positions and is a Gaussian centered at the targe location $\varvec{x}'$ with inverse covariance matrix *S*, i.e., $Q(\varvec{x},\varvec{x}') = Q(\varvec{x}-\varvec{x}') \sim N(\varvec{x}',S^{-1})$. The results in the commonly used Gaussian regularization kernel. Generally, however, more complex coupling schemes can incorporate more relevant prior information, resulting in more robust warping schemes. For more details, the reader is referred to^43^.

**Figure 15:**
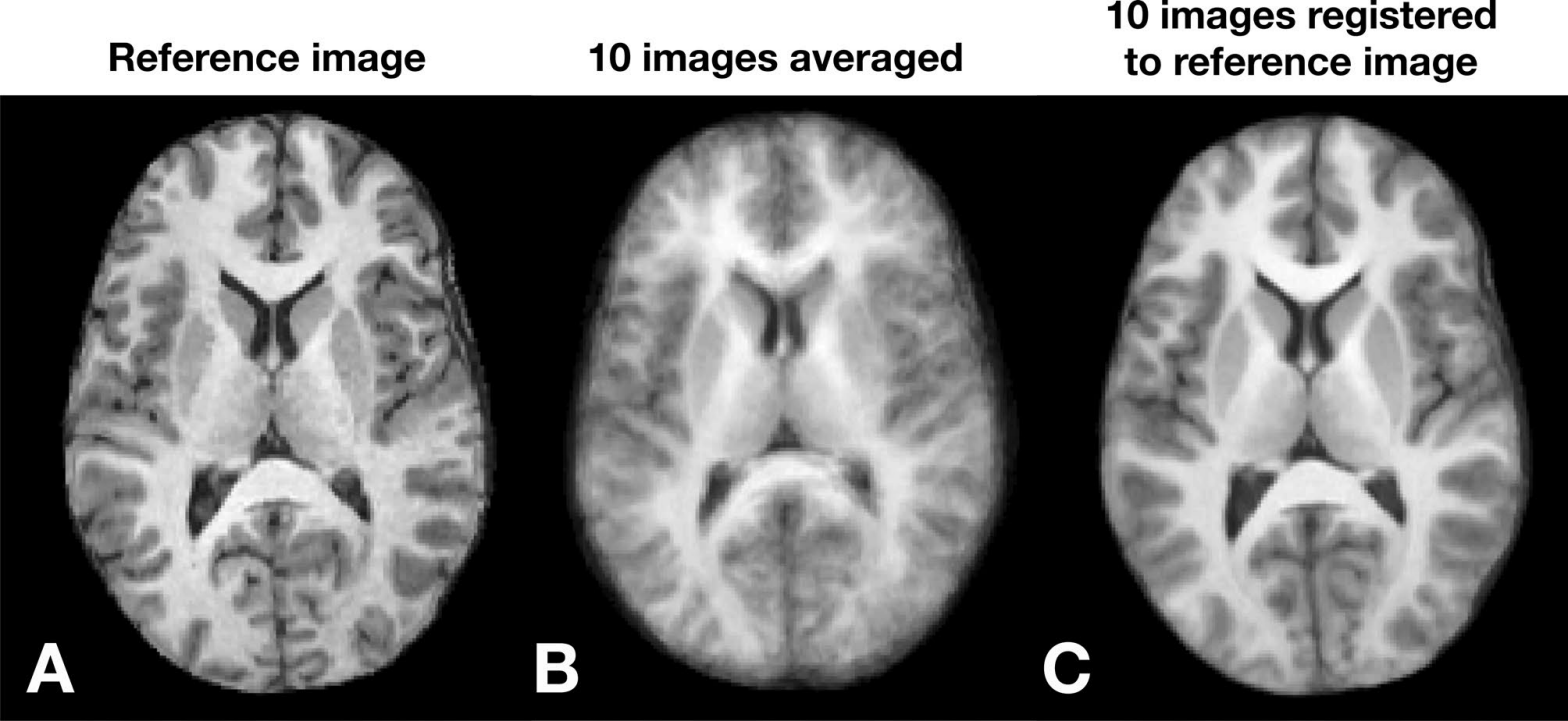
Registration of multiple high resolution anatomical (HRA) MRI scans using symplectomorphic registration (SYMREG). (**A**) Single reference HRA, (**B**) average of HRA datasets from 10 different people. Blurring is do to subtle natural shape variations between individual subjects (**C**) average of 10 images in (**B**) after registration with template image in (**A**) from (adapted from^43^).

**Figure 16:**
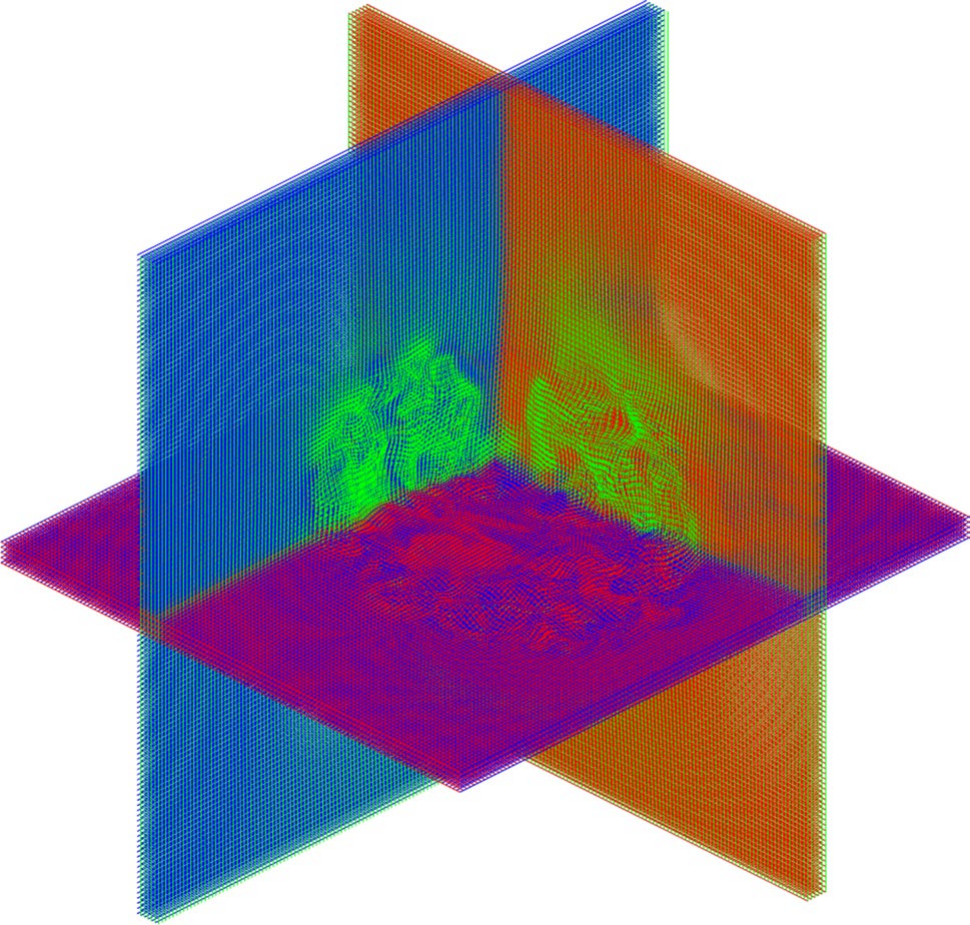
Actual 3D volumetric SYMREG warping grid used in the registration of Fig. 15.

An example of the use of SYMREG registration on multiple MRI volumetric brain images from different normal human volunteers is shown in Fig. 15^43,89^. This demonstrates that naïvely combining (e.g., averaging) datasets (e.g., brains) from multiple subjects produces highly blurred images due to the natural geometric variations of the organs between individuals (Fig. 15B). Accurately taking into accounts the non-linear geometrical relations using SYMREG produced a combined image with little blurring Fig. 15C). The corresponding volumetric 3D warping grid used in the registration in Fig. 15 generated by SYMREG is shown in Fig. 16.

In summary, SYMREG allows for rapid and robust automated non-linear registration of multi-modality multi-subject data.

**Table 1:** CT scan datasets used in this study deposited in MorphoSource.org.

Figure	Group/sample	Specimen$\sharp$	Description/element	taxon	Doi link
1	27 days old	TMM:M:7595	Skull	*Monodelphis domestica*	10.17602/M2/M168916
1	48 days old	TMM:M:7536	Skull	*Monodelphis domestica*	10.17602/M2/M168701
1	57 days old	TMM:M:7539	Skull	*Monodelphis domestica*	10.17602/M2/M168773
1	75 days old	TMM:M:7542	Skull	*Monodelphis domestica*	10.17602/M2/M168829
1	90 days old	TMM:M:7545	Skull	*Monodelphis domestica*	10.17602/M2/M168884
1	Retired breeder	TMM:M:7599	Skull	*Monodelphis domestica*	10.17602/M2/M168948
2 and 4	“Anthropoid”	MCZ:mamm:10132	Cranium	*Saimiri oerstedii*	10.17602/M2/M4489
2 and 4	“Anthropoid”	MCZ:mamm:10133	Cranium	*Saimiri oerstedii*	10.17602/M2/M4488
2 and 4	“Anthropoid”	MCZ:mamm:27098	Cranium	*Cebus apella*	10.17602/M2/M5207
2 and 4	“Anthropoid”	MCZ:mamm:29488	Cranium	*Saimiri oerstedii*	10.17602/M2/M4484
2 and 4	“Anthropoid”	MCZ:mamm:30724	Cranium	*Cebus apella*	10.17602/M2/M5210
2 and 4	“Anthropoid”	MCZ:mamm:34326	Cranium	*Cebus capucinus*	10.17602/M2/M5148
2 and 4	“Anthropoid”	MCZ:mamm:43484	Cranium	*Saimiri sciureus*	10.17602/M2/M4445
2 and 4	“Strepsirrhine”	AMNH:mammals:M-100640	Cranium	*Cheirogaleus major*	10.17602/M2/M12868
2 and 4	“Strepsirrhine”	AMNH:mammals:M-170553	Cranium	*Lepilemur leucopus*	10.17602/M2/M11537
2 and 4	“Strepsirrhine”	AMNH:mammals:M-170558	Cranium	*Lepilemur leucopus*	10.17602/M2/M11533
2 and 4	“Strepsirrhine”	AMNH:mammals:M-170561	Cranium	*Lepilemur leucopus*	10.17602/M2/M11666
2 and 4	“Strepsirrhine”	AMNH:mammals:M-170568	Cranium	*Lepilemur mustelinus*	10.17602/M2/M11664
2 and 4	“Strepsirrhine”	AMNH:mammals:M-170573	Cranium	*Lepilemur leucopus*	10.17602/M2/M11662
2 and 4	“Strepsirrhine”	AMNH:mammals:M-196618	Cranium	*Cheirogaleus major*	10.17602/M2/M23465
3	na	AMNH:mammals:M-72141	Cranium	*Callicebus cupreus*	10.17602/M2/M21971
3	na	AMNH:mammals:M-72143	Cranium	*Callicebus cupreus*	10.17602/M2/M21972
5	Flexed/treeshrew	FMNH:mammals:76865	Flexed hind limb	*Tupaia minor*	10.17602/M2/M30401
5	Extended/treeshrew	FMNH:mammals:76865	Extended hind limb	*Tupaia minor*	10.17602/M2/M30398
5	Flexed/primate	DPC:ost:030	Flexed hind limb	*Microcebus murinus*	10.17602/M2/M30424
5	Extended/primate	DPC:ost:030	Extended hind limb	*Microcebus murinus*	10.17602/M2/M30420
6	na	FMNH:PR:2081	Cranium& hemi-mandible	*Tyrannosaurus rex*	10.17602/M2/M366912

## Data Availability

Researchers interested in obtaining the SAPID software can contact Dr. Lawrence Frank at lfrank@ucsd.edu. Data used in this study is listed in Table 1 which includes the Digital Object Identifiers (DOI) where users can access the data.
